# Cyclic-FMN Is a Detectable, Putative Intermediate of FAD Metabolism

**DOI:** 10.3390/biom16010175

**Published:** 2026-01-21

**Authors:** Luxene Belfleur, Juha P. Kallio, Wito Richter, Natalie R. Gassman, Mathias Ziegler, Marie E. Migaud

**Affiliations:** 1 Mitchell Cancer Institute, Mass Spectrometry Core Facility, University of South Alabama, Mobile, AL 36604, USA; lbelfleur@southalabama.edu; 2 Department of Biomedicine, University of Bergen, Jonas Lies vei 91, 5009 Bergen, Norway; juha.kallio@bluehelixhealth.com (J.P.K.); mathias.ziegler@uib.no (M.Z.); 3 Department of Biochemistry, University of South Alabama, Mobile, AL 36688, USA; richter@southalabama.edu; 4 Department of Pathology, Heersink School of Medicine, University of Alabama at Birmingham, Birmingham, AL 35294, USA; nrg2@uab.edu; 5 Department of Pharmacology, F. P. Whiddon College of Medicine, University of South Alabama, Mobile, AL 36688, USA

**Keywords:** vitamin B2, riboflavin, FAD, cyclic-FMN, cyclic nucleotide, divalent cations

## Abstract

Free flavin adenine dinucleotide (FAD) is metabolized to flavin mononucleotide (FMN) and adenine monophosphate (AMP) by hydrolases and to 4′,5′-cyclic phosphoriboflavin (cFMN) and AMP by the triose kinase FMN cyclase (TKFC). Yet, the lack of analytical standards for cFMN might have resulted in the incidence of cFMN in biological specimens being underreported. To address this shortcoming, cFMN was synthesized from either FMN or FAD. The optimization of the FAD to cFMN reaction conditions revealed that an equimolar ratio of ZnSO_4_ and FAD yielded pure cFMN upon the precipitation of AMP-Zn salts. cFMN is stable to aqueous acidic and basic conditions and is readily extracted from biological samples for detection by liquid chromatography coupled with mass spectrometry. Although cFMN is hydrolyzed by liver tissue extracts to FMN and riboflavin, the mechanisms for this conversion remain elusive.

## 1. Introduction

**Figure 1:**
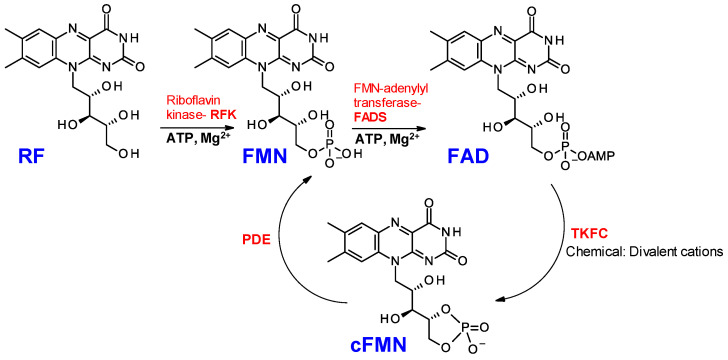
Biosynthetic pathway of riboflavin-derived cofactors, FMN, FAD, and cFMN. RF: riboflavin; FMN: flavin mononucleotide; FAD: flavin adenine dinucleotide; cFMN: 4′,5′-cyclic-phosphoriboflavin; RFK: riboflavin kinase; FADS: flavin adenine dinucleotide synthase; TKFC: triose kinase/flavin cyclase; PDE: phosphodiesterase. Note: Scheme S1 in the Supplementary Materials illustrates the mechanistic nuances that define the FAD to FMN interconversion via cFMN.

Riboflavin (vitamin B2) is an essential nutrient in mammals, and the cofactors derived from riboflavin are considered essential for all known life forms on Earth. Once converted into its coenzyme forms, riboflavin is critical to virtually all major energy-producing metabolic pathways of mammalian biology, catalyzing more than eighty enzymatic reactions involving one-electron and two-electron transfer in cells [1,2,3,4,5]. In short, riboflavin-dependent cofactors are critical to mitochondrial respiration (Complex I and II), one-carbon pathway and folate homeostasis, and fatty acid beta oxidation, to name only a few of the bioenergetic and biosynthetic pathways that rely on riboflavin-derived cofactors. Imbalances in riboflavin metabolism are associated with neurological diseases [6], amino acid and fatty acid metabolism disorders [7], and finally diseases associated with mitochondrial dysfunction, such as complex I deficiencies and Leigh Syndrome [8]. To be functional, riboflavin is converted to flavin mononucleotide (FMN) by riboflavin kinase (RFK) and subsequently converted to flavin adenine dinucleotide (FAD) by FAD synthase (FADS) [9,10,11,12,13] (Figure 1). This biosynthetic sequence is reminiscent of that of nicotinamide riboside, a vitamin B3 component, which is converted to nicotinamide adenine dinucleotide (NAD) [14,15,16,17,18]. Notably, in addition to NAD, the reduced form of NAD, NADH, and the phosphorylated form, NADP(H), are frequent partners of FMN and FAD in redox biology [10,19,20,21,22,23,24]. Due to the central role of FMN and FAD in cellular homeostasis and metabolism, a deeper understanding of FAD metabolism, metabolic regulation, biosynthesis, and degradation is warranted.

Light sensitivity often plagues the handling of FMN and FAD [25] in solution, where FAD is also prone to hydrolysis. In cells, the FMN portion of FAD can be transferred onto proteins by FMN-transferases or released from FAD by hydrolases such as NUDIX [11] and ApbE-like [26] enzymes. FAD can also convert to 4′,5′-cyclic phosphoriboflavin (cFMN), as first reported by Fraiz, who detected a low K_m_ FMN cyclase in rat liver extracts [27]. Since then, the conversion of FAD into cFMN and AMP has been shown to be catalyzed by the triose kinase/flavin cyclase (TKFC) [27] (Figure 1). TKFC, also known as dihydroxyacetone kinase (DAK), is a calcium/ATP-dependent kinase central to triose metabolism [28,29]. Despite the extensive knowledge acquired on the functionalization of trioses by TKFC, the biological significance of TKFC FAD-cyclase activity and cFMN remains unexplained.

Overall, the chemical and analytical tools used to report on cFMN formation and function have been limited. FAD, FMN, and FAD hydrolysis are most often monitored using fluorescence, since FAD and FMN/cFMN possess different spectral properties. Yet, cFMN and FMN cannot be differentiated by direct fluorescence measurements. Finally, although zinc-based artificial FMN/FAD chemosensors that capitalize on the structural differences between FMN and cFMN have been developed [30], no report of this sensor’s use appears in the literature. As a result, cFMN might have been misidentified as FMN in biospecimens. This work reports using a facile chemical method for generating cFMN free of FMN and FAD, and also uses an analytical method that combines liquid chromatography with mass spectrometry (LC-MS) to differentiate between cFMN, FMN, and FAD. The account also provides the first evidence that cFMN is an FAD metabolite that is biochemically hydrolyzed to riboflavin by tissue extracts.

## 2. Materials and Methods

Materials: Flavin adenine dinucleotide disodium salt hydrate (FAD, solid powder) and riboflavin 5′-monophosphate sodium salt (FMN, solid powder) were purchased from AK Scientific (Union city, CA, USA) and TCI (Portland, OR, USA) respectively. Ammonia (4% in ethanol) and trifluoroacetic anhydride were purchased from TCI (Portland, OR, USA) and Alfa-Aesar (Ward Hill, MA, USA), respectively. Calcium sulfate (solid powder) and zinc sulfate (solid crystalline) were purchased from J.T. Baker (Phillipsburg, NJ, USA) and Mallinckrodt Baker (Phillipsburg, NJ, USA), respectively. Calcium carbonate (solid powder) and sodium chloride (solid crystalline) were purchased from Fisher Scientific (Ward Hill, MA, USA). Magnesium chloride hexahydrate (solid crystalline) was purchased from Alfa-Aesar (Ward Hill, MA, USA), and copper (II) chloride or copper-trace (solid crystalline) was purchased from Arctom Chemicals (Westlake Village, CA, USA). Potassium chloride (solid crystalline), HEPES (solid powder), and trimethyl phosphate (TMP, liquid) were purchased from Sigma-Aldrich (St Louis, MO, USA). Tris-HCl (0.5 M, pH 6.8) was purchased from ThermoFisher (Ward Hill, MA, USA), and 10× PBS (containing 1.37 M NaCl, 0.027 KCl, and 0.119 M phosphate) was purchased from Fisher Scientific (Ward Hill, MA, USA). All the reagents were used without further purification.

Characterization: ^1^H and ^31^P NMR spectra were recorded on a Bruker Avance III HD 400 spectrometer (TopSpin 3.5 pl 6, Billerica, MA, USA) instrument with nominal frequencies of 400.1 MHz for proton and 162 MHz for phosphorus. Deuterium oxide (purchased from ACROS Organics, Morris plains, NJ, USA) and trimethyl phosphate were used as internal references. ^1^H NMR chemical shifts are reported in parts per million (ppm). The NMR experiments were performed in buffered solutions (50 mM HEPES or Tris-HCl, 90%) containing D_2_O (10%) with 5 mM TMP at 298.2 K.

Buffers’ preparation and composition: The Tris-HCl buffer solution (50 mM) was prepared by diluting 0.5 M Tris-HCl and adjusting the pH (7.4 and 8) using a NaOH solution (200 mM). The HEPES buffer (50 mM) was prepared by dissolving the solid powder in distilled water and by adjusting the pH to pH 7.4 or pH 8 with an aqueous NaOH solution (200 mM).

Tissue extracts: Tissues were obtained as waste samples from control, non-treated animals. The harvested tissues were immediately flash-frozen upon collection and stored at −80 °C until use for these experiments.

General Procedures:

Synthesis of 4′, 5′-cyclic riboflavin 5′-mononucleotide (cFMN) from FMN.

In a vial (20 mL), 1 g of riboflavin 5′-monophosphate sodium salt (2.09 mmol; 1 eq.) was mixed with 3.8 mL of trifluoroacetic anhydride (27.17 mmol; 13 eq.) on a stirrer plate at room temperature for 24 h according to Nagelschneider et al. [31]. The excess of trifluoroacetic anhydride was removed under reduced pressure, and an oily residue was obtained. The crude material was resuspended in dry ethanol (30 mL), and the mixed anhydride intermediate was precipitated with diethyl ether (40 mL). The suspension was filtered, and a yellow solid was recovered. Ethanolic ammonia (50 mL) was added to the solid, and the suspension was stirred for 5 h before being filtered to isolate a yellow solid. Diethyl ether and ethanol were removed under reduced pressure over two days. A total amount of 905 mg of a mixture of cFMN and FMN was obtained. NMR analysis (^1^H and ^31^P-NMR) of this mixture in D_2_O revealed a 46% conversion of FMN into cFMN according to this protocol.

Synthesis of 4′, 5′-cyclic riboflavin 5′-mononucleotide (cFMN) from FAD.

In a vial, FAD (49.70 mg, 1 equiv. 0.06 mmol) was mixed with CaSO_4_ or ZnSO_4_ (1 equiv.) in Tris-HCl (6 mL, 50 mM, pH 7.4). The reaction mixture was heated in an oil bath (37 °C) with stirring for 24 h. cFMN and AMP formed quantitatively. However, when ZnSO_4_ was used instead of CaSO_4_, AMP-Zn salt precipitated, while cFMN salt remained in solution. The cFMN solution was obtained following the centrifugation and removal of the zinc salt of AMP. cFMN was found to be >90% pure by ^1^H-NMR, and ^31^P-NMR, and possible contamination by AMP and FAD was measured by UV spectrometry at 260 nm (λ_max_ of AMP and FAD at 260 nm). Chelex-100 resin (200–400 mesh, sodium form) from BIO-RAD Laboratories Ltd (Hercules, California, US) was used to remove the divalent cations (Ca^2+^ or Zn^2+^) and form the free acid form of cFMN. ^1^H NMR (400 MHz, D_2_O): δ 7.73 (s, ^1^H), 7.61 (s, ^1^H), 5.07 (dd, j = 12 Hz, j = 16 Hz, ^1^H), 4.75 (d, j = 16 Hz, ^1^H), 4.71 (m, ^1^H), 4.47 (m, ^1^H), 4.32 (m, ^2^H), 4.13 (m, ^1^H), 2.53 (s, ^3^H), 2.39 (s, ^3^H). ^13^C NMR (100 MHz, D_2_O): δ 162.40, 159.22, 152.17, 151.32, 140.83, 135.72, 135.32, 132.94, 13.78, 118.26, 76.80, 74.03, 70.83, 67.89, 49.07, 22.22, 20.04. ^31^P NMR (162 MHz, D_2_O): δ 17.96. HRMS calculated for C_17_H_19_N_4_O_8_P[M]+ 439.10132 found 439.10238; UV: λ_max_ 445 nm and ε = 12,200 M^−1^·cm^−1^

Evaluations of the effects of salt, pH, buffer type, and temperature: In separate vials, FAD (4.97 mg, 1 equiv. 0.006 mmol) was mixed with one of the following metals cations (1 equiv. 0.006 mmol): NaCl, KCl, MgCl_2_·6H_2_O, CaSO_4_, ZnSO_4_.7H_2_O and CaCO_3_ in Tris-HCl, HEPES (0.6 mL, 50 mM, pH 7.4 or pH 8), or PBS (0.6 mL, 1×, pH 7.4 or pH 8). The reaction mixtures were heated in an oil bath to 37 °C or 50 °C with stirring for 3, 6, or 24 h.

Dose–response experiments: FAD (10 mM) was mixed in Tris-HCl (50 mM, pH 7.4 or pH 8) with several concentrations of CaSO_4_ (0 nM, 100 nM, 1 µM, 10 µM, 100 µM, 1 mM, 3 mM, 6 mM, and 9 mM) prepared by serial dilution from a stock solution of CaSO_4_ (10 mM).

Stability under acidic and basic conditions: cFMN (10 mM) was dissolved in 0.1 M HCl and 0.1 M NaOH (10% D_2_O) and incubated for 24 h at rt and 37 °C. Surprisingly, no product degradation was observed by ^1^H-NMR and ^31^P-NMR at either pH.

LC-MS conditions of FAD, FMN, and cFMN:

An Agilent 1200 Series HPLC coupled to a Thermo Q-Exactive Plus mass spectrometer was used for sample analysis. An autosampler was used to inject 2 µL of each sample onto the analytical column (2.1 × 150 mm, 5-micron 200 Å HILICON iHILIC^®^-Fusion(P); Waters (Milford, Massachusetts, USA)), guarded by a pre-column (2.1 mm × 20 mm, 200 Å HILICON iHILIC^®^- Fusion(P) Guard Kit) operating at room temperature. Solvent A consisted of 10 mM ammonium acetate in H_2_O at pH ~9, and Solvent B consisted of Acetonitrile. A flow rate of 100 µL per minute was used for the first 0.5 min of the run at 90.0% B solvent, followed by a gradient from 90.0 to 50.0% B from 0.5 to 20 min, along with an increased flow rate of 200 µL per minute. The solvent was held at 50.0% B from 20 to 21 min and returned to 90.0% B from 21 to 29 min at the same flow rate to re-equilibrate the column, then remained at 90.0% B from 29 to 30 min at a flow rate of 100 µL per minute for the remainder of the run. A HESI (heated electrospray ionization) source was used in positive polarity, with a capillary temperature of 320 °C, a source voltage of 3.2 kV, an S-lens RF level of 60, and a sheath gas flow rate of 16.0. One full scan from 100–900 *m*/*z* was performed at 17,500 resolution, followed by targeted MS^2^ scans of FAD (786.16441 *m*/*z*), FMN (457.11190 *m*/*z*), and cFMN (439.10130 *m*/*z*) at 35,000 resolution with an isolation width of 2.0 *m*/*z*. Normalized CID collision energy was set to 25. The total run time was 38 min. A blank was run in between each sample to minimize and monitor for carryover.

LC-MS conditions of FAD, FMN, and cFMN as standards and measured in whole blood:

5 nmol of FAD or cFMN was added to 200 µL of whole blood in a 1.5 mL centrifuge tube on ice. Then, 400 µL of degassed, cold MeOH (−80 °C) was added to each tube containing the blood sample spiked with FAD or cFMN. The samples were sonicated at 4 °C (3×) for 10 sec each. Afterwards, 400 µL of degassed CHCl_3_ (−80 °C) was added to each sample in the tube and vortexed. 200 µL of degassed water (4 °C) was then added to each sample, and the solution was vortexed. The samples were centrifuged at 13,000 rpm for 15 min at 4 °C. Carefully, 400 µL of the top aqueous phase containing the metabolites was transferred to a 2 mL Eppendorf tube. The supernatants were frozen in liquid nitrogen and freeze-dried on a lyophilizer. Each dried sample was resuspended in 30 µL of buffer A2 (10 mM ammonium acetate at pH 9) and analyzed by LC-MS. 200 µL of blood not spiked with FAD or cFMN was extracted as a control. In addition, 5 nmol of FAD and cFMN in an equivalent volume of buffer were extracted and analyzed separately according to the same protocol, while 5 nmol of FAD and cFMN (non-extracted) were freeze-dried, resuspended in 30 µL of buffer A2, and analyzed by LC-MS.

Tissue homogenization protocol for PDE assay: Tissue samples (73–315.2 mg) previously frozen were placed in an ice bath. The samples were transferred into pre-cooled (ice) 2 mL homogenization tubes containing ceramic beads (2.8 mm diameter). Pre-cooled buffer (50 mM Tris-HCl, 12 mM MgCl_2_, 100 mM NaCl, 1 mM dithiothreitol, and pH 7.5, 0 °C) was added to the samples (1.1 μL/mg for liver and brain tissue, 1.5 μL/mg for kidney and lung tissue, and 2.1 μL/mg for heart tissue). The tissues were then homogenized for 30 s (3×) at 4800 rpm, with 30 s intervals between the homogenization steps. Afterwards, the tubes were centrifuged for 5 min at 1000 rpm at 4 °C, then the supernatants were transferred to 1.5 mL Eppendorf tubes, followed by a second centrifugation for 10 min at 13,000 rpm at 4 °C. The supernatants were transferred to new 1.5 mL Eppendorf tubes and placed on ice for use in the following assays.

Hydrolysis of FAD and cFMN by PDE present in mouse tissue extracts in presence or absence of Ca^2+^: In an NMR tube, FAD (10 mM) or FAD (10 mM) and CaSO_4_ (10 mM), or cFMN (10 mM), HEPES buffer (50 mM, pH 7.4), 10% D_2_O and mouse tissue extract resuspended in buffer (50 µL) were mixed for a total final volume of 500 µL. The samples were incubated at 37 °C in an oil bath and monitored regularly by ^31^P NMR (ns = 40) at 30 min intervals for 2 h and after 24 h. For t = 0, two NMR spectra were acquired. The first spectrum was acquired before the tissue extract and CaSO_4_ salts were added, and the second just after the addition of the extract and the salt.

Mouse tissue extracts hydrolysis of cFMN and cAMP: In an NMR tube, cFMN (2 mM or 10 mM) or cAMP (2 mM), trimethyl phosphate (2 mM), Tris-HCl buffer (50 mM, pH 7.5), 10% D_2_O, and mice tissue extract (2.5 mg/mL) were mixed to a final volume of 500 µL. The samples were incubated at 37 °C in an oil bath and monitored regularly by ^31^P NMR (ns = 40) at 30 min or 1 h intervals. At t = 0, NMR spectra were acquired before adding the tissue extract and just after adding the tissue extract. The composition of the buffer used for this set of experiments is Tris-HCl (50 mM), MgCl_2_ (12 mM), NaCl (100 mM), dithiothreitol (1 mM), pH 7.5.

CNPase activity: Mouse CNPase (kindly provided by Professor Petri Kursula, University of Bergen) was used to determine whether cFMN serves as a substrate for CNPase. The cleavage of cNADP was used as a positive control to verify enzyme activity. The control reaction containing 250 µL of 25 mM Tris buffer, pH 7.4, 2 mM cNADP, and 5 µg of CNPase enzyme was incubated for 90 min at 20 °C. The reaction containing cFMN instead of cNADP was set up the same way, except with an overnight incubation (20 h). In addition, another reaction with 2 mM cFMN and 15 µg of CNPase was performed at 37 °C. After the reaction, samples were filtered using Vivaspin centrifugal concentrators (5 kDa cutoff) to remove the protein and any precipitate. Analytes were diluted to ~60 µM for MS analysis for the detection of the hydrolyzed products.

## 3. Results

FAD, FMN, and cFMN can be differentiated by nuclear magnetic resonance (NMR) spectroscopy and mass spectrometry (MS), but both techniques require chemical standards to facilitate detection and quantification. In the attempted synthesis of FAD from FMN catalyzed by divalent cations under mechanochemical conditions [32], cFMN formed readily as revealed by ^31^P-NMR spectroscopy. It was also observed that although FAD production plateaued early, FMN continued to be consumed, while cFMN and AMP became the predominant species in the reaction mixture as long as divalent cations were present. Similarly, no cFMN formed from FMN in the absence of activated-AMP. These observations led us to conclude that the divalent cations promoted the conversion of FAD to cFMN (Figure 1). Building on these latter observations, we report an effective synthesis of cFMN, reveal the chemical and enzymatic stability of cFMN, and provide evidence that liquid chromatography coupled with mass spectrometry (LC-MS) can be used with confidence to detect FAD, FMN, and cFMN in biological samples.

### 3.1. Non-Enzymatic cFMN Formation and Conditions of Cyclisation

**Figure 2:**
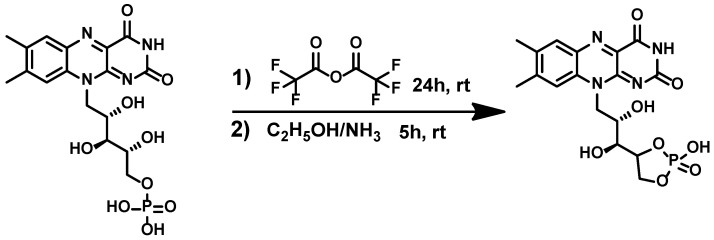
Chemical synthesis of cFMN from FMN.

**Figure 3:**
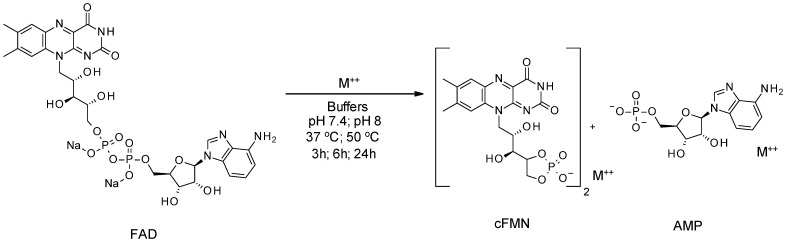
Chemical synthesis of cFMN from FAD.

cFMN can be generated from FMN (free acid) (Figure 2) via a mixed anhydride intermediate [31]. Unfortunately, this synthesis is low-yielding and requires extensive purification. As an alternative method, Pinto and coworkers reported the formation of cFMN from FAD using MnCl_2_ in buffer [27] (Figure 3). Although slightly higher yielding (~20%), this approach also requires additional purification to remove AMP and unreacted FAD, and to isolate pure cFMN.

**Figure 4:**
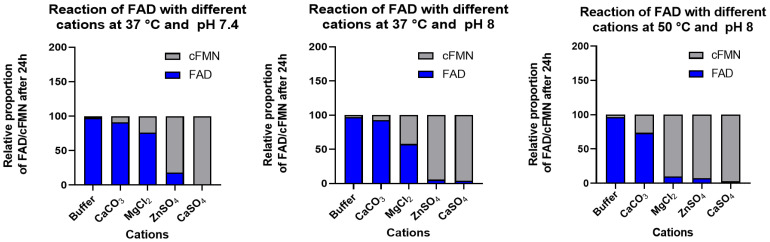
FAD ([FAD] = 10 mM) conversion to cFMN in Tris-HCl (50 mM) catalyzed by molar equivalency of divalent cations, reported as a function of pH, and temperature after 24 h (^1^H and ^31^P-NMR spectra can be found in the Supplementary Materials, Figures S1–S6).

Given our observations obtained under mechanochemical conditions, we anticipated that the synthesis of cFMN from FAD could be optimized to achieve better conversion and isolated yields. We tested monovalent and divalent cations and identified the cations that allowed the highest FAD to cFMN conversion (Figure 4; Table 1). We then investigated whether buffers (Tris-HCl, HEPES, or PBS) and temperatures (37 °C and 50 °C) could be optimized (Table 1). Ultimately, molar equivalents of divalent salts added to a solution of FAD, dissolved in a non-phosphate-containing buffer, pH 8, at 50 °C, yielded the best conversion of FAD to cFMN and AMP, with no FMN formation (Figure 4).

The formation of cFMN and AMP, monitored by ^31^P-NMR and confirmed by ^1^H-NMR, was observed when FAD was exposed to MgCl_2_, ZnSO_4_, and CaSO_4_ (Table 1), but absent when monovalent cations (K^+^ and Na^+^) were applied (Figures S7 and S8). A puzzling reaction outcome was the low conversion observed when CaCO_3_ was used as the source of divalent cation. However, this observation can be attributed to the low dissociation constant of CaCO_3_ in water. Unlike the other salts, CaCO_3_ is only partially soluble in non-acidic buffers (e.g., solubility at pH 7: CaSO_4_ 19.3 mmol/L vs. CaCO_3_ 0.47 mmol/L), releasing lower levels of free Ca^2+^ in solution than CaSO_4_ [33]. The reduced availability of free calcium ions due to the reduced solubility of CaCO_3_ is likely responsible for the low conversion of FAD to cFMN. However, at higher temperatures, the solubility of CaCO_3_ increases, as does the amount of cFMN formed. Together, these results point to the need for free divalent cations in the present solution in a sufficient concentration to enable the conversion. Upon ^31^P-NMR reaction monitoring, we observed that FAD was almost totally consumed within 3 h when the reaction was conducted with molar equivalents of CaSO_4_ in HEPES at 37 °C. Such a conversion was prevented when PBS was used for buffering the reaction (Table 1; Figures S9–S16). In PBS, inorganic phosphate complexes calcium ions [34]. This further confirms our premise that free divalent cations are needed for the conversion. Overall, Ca^2+^ proved to be the most efficient cation for forming cFMN from FAD (Table 1). Interestingly, Mg^2+^, a common cofactor of ATP-dependent enzymatic processes, was a much weaker cyclization catalyst than Ca^2+^ and Zn^2+^.

The reactions of FAD with divalent cations generated approximately a 1/1 ratio of cFMN and AMP, except for Zn^2+^ where 86% of cFMN (^31^P NMR δ 17.9 ppm) and 8% of AMP (^31^P NMR δ ~ 4 ppm) were detected by ^31^P NMR after 3 h in HEPES at pH 8 and 37 °C (Figure S9). Ultimately, both the ^31^P-NMR FAD and AMP signals disappeared, and a solid suspension appeared in the NMR tube. This suspension was centrifuged, and the solid was identified as ZnAMP by ^1^H-NMR, ^31^P-NMR, and LC-MS. (cFMN)_2_Zn remained in the supernatant free of AMP and FAD by ^1^H-NMR and ^31^P-NMR (>98%) and required little to no purification.

We then examined the effect of the molar equivalency of divalent cation to FAD to evaluate whether sub-molar equivalencies of divalent cations could be used for the complete conversion of FAD to cFMN. To a 10 mM solution of FAD in Tris-HCl buffer (50 mM, pH 7.4) at 37 °C, the sub-molar equivalency of cations led to the partial conversion of FAD to cFMN and AMP (Supplementary Materials Figure S17), with the first sign of cFMN formation occurring at 1 μM CaSO_4_ to 10 mM FAD equivalency (Supplementary Materials, Figures S18 and S19). This observation could be biologically relevant as Ca^2+^ concentration can fluctuate significantly in organelles like the mitochondrion, where FAD is most abundant [11,35,36,37].

### 3.2. Characterization of the Elution Profile of FAD, FMN, and cFMN Present in Standard Solutions by LC-MS and In-Source Fragmentation

**Figure 5:**
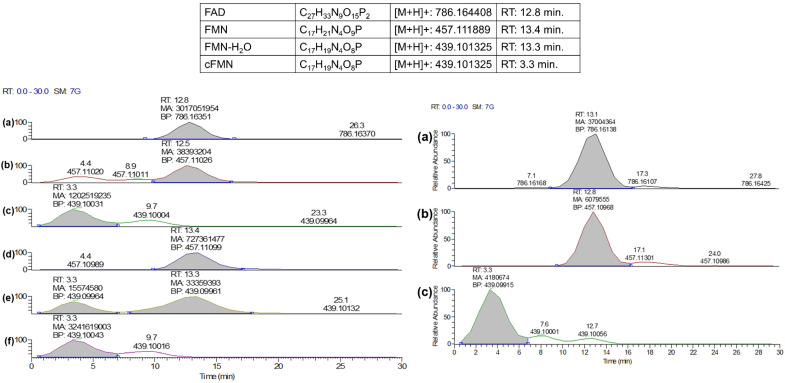
Left panel: Stacked (HILIC) LC-MS spectra: 5L-(**a**) “pure” FAD (100 µM) in solution. 5L-(**b**) FMN trace from the decomposition of FAD in solution and LC-elution and FMN from FAD in-source fragmentation. 5L-(**c**) cFMN trace from the decomposition of FAD in solution. 5L-(**d**) FMN standard (100 µM) in solution. 5L-(**e**) cFMN detected in the commercial FMN solution (RT: 3.3 min) and FMN-H_2_O (amu 18) from in-source fragmentation caused by a loss of water (RT: 13.3 min), and 5L-(**f**) synthetic cFMN (100 µM) standard. Right panel: Stacked HILIC LC-MS spectra of 5R-(**a**) FAD, 5R-(**b**) FMN, and (**c**) 5R-cFMN detected in mouse heart tissue. Note: In-source fragmentation of FAD to cFMN is not observed in 5L-(**c**). The abundance observed in 5R-(**c**) at 12.7 min may be in-source cFMN formed from FMN or dehydration of FMN (FMN-H_2_O).

By gaining access to riboflavin-derived chemical standards, we confirmed that the species sharing retention time, *m*/*z* ion mass, fragmentation pattern, and molecular formula with FAD, FMN, and cFMN were easily detected in biological extracts when these were analyzed by liquid chromatography coupled with mass spectrometry, i.e., LC-MS/MS (see experimental section for details) (Figure 5). However, regardless of the chromatographic method applied, we were unable to separate FMN from FAD. This is a limitation since in-source FAD conversion to cFMN and FMN may occur.

### 3.3. Detection of FAD, FMN, and cFMN Present in Blood Samples

Therefore, we then considered the risks of in-source FAD fragmentation and the artifactual formation of cFMN and FMN. The analyses of pure FAD, FMN, and cFMN by HILIC-LC-MS/MS (Figure 5L-a,d,f) revealed that FAD fragmentation to AMP and FMN occurred readily in-source (Figure 5L-b), while we did not observe cFMN generated from in-source fragmentation of FAD (Figure 5L-c). However, we detected cFMN at 3.3 min in the standard solution of FMN, along with a peak with the same retention time as FMN of 13.3 min, exhibiting an *m*/*z* that could correspond to the in-source fragmentation of FMN to cFMN or another dehydrated FMN fragment (Figure 5L-e).

**Figure 6:**
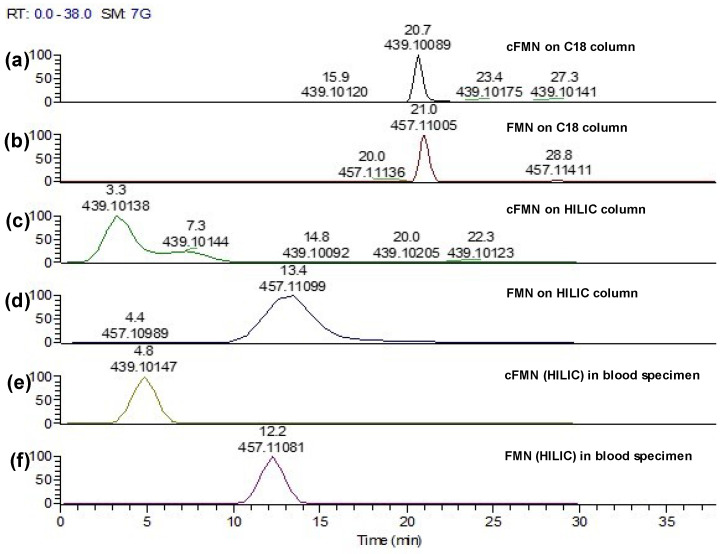
C18 LC-MS traces of: (**a**) cFMN standard; (**b**) FMN standard, and HILIC LC-MS traces of: (**c**) cFMN standard; (**d**) FMN standard; (**e**) cFMN in blood specimen; (**f**) FMN in blood specimen. Note: The extraction protocol uses cold methanol, and chloroform is not buffered. This can cause chromatographic shifts and different retention times for cFMN between standards and blood-extracted samples. This is particularly prevalent here since cFMN ionization status can vary substantially because of it being a cyclic phosphate monoanionic species with pKa~6.

To examine whether a different chromatography method could help resolve this latter observation, we applied reverse-phase conditions on a C18 column. Unfortunately, FMN and cFMN could not be readily resolved under these conditions (Figure 6a,b). Overall, a better separation of cFMN from FAD and FMN was achieved when using HILIC conditions (Figure 5L-d,f). Having the chromatographic conditions that separate cFMN from FAD and FMN, we are now able to differentiate the naturally occurring cFMN present in specimens from the cFMN generated from in-source fragmentation of FMN or on-column from FAD.

However, when implementing our LC-MS/MS method to detect FAD, FMN, and cFMN in biospecimens such as blood, we had to consider the possibility that the extraction protocol might promote the conversion of FAD to cFMN. To evaluate this possibility, we first analyzed whether cFMN could be detected in blood and whether the in-source FMN to cFMN conversion affected the quantification (Figure 6). Following sample extraction, cFMN and FMN are readily differentiated (Figure 6c–f) when using a HILIC instead of a C18 column for the LC-MS separation. It should be noted that although FMN has a retention time of ~12 min, it is also detected at ~5 min, the retention time corresponding to cFMN. Therefore, it is also likely that cFMN is hydrated in-source to FMN before its detection at that retention time.

We then analyzed the composition of free FAD, FMN, and cFMN in blood by LC-MS, before and after spiking the blood sample with FAD or cFMN. In cells, riboflavin-derived cofactors can either be free or protein-bound. Only the quantification of free riboflavin-derived cofactors can be achieved by LC-MS following the sample extraction process, since the riboflavin-derived cofactors that are covalently bound to proteins precipitate out of solution with their partnered proteins. The measurements of FMN and FAD in biospecimens are challenging and mostly use fluorescence, with EGRAC (Erythrocyte Glutathione Reductase Activation Coefficient) being traditionally used to assess riboflavin status. However, in erythrocytes, the median concentrations of FMN and FAD have been reported to be 44 and 469 nM, respectively [38], while concentrations in plasma are reported to be ten-fold lower [39]. A more recent clinical development has led to more robust spectrophotometric protocols that seek to directly measure free FMN [40]. However, these protocols cannot differentiate between cFMN and FMN.

**Figure 7:**
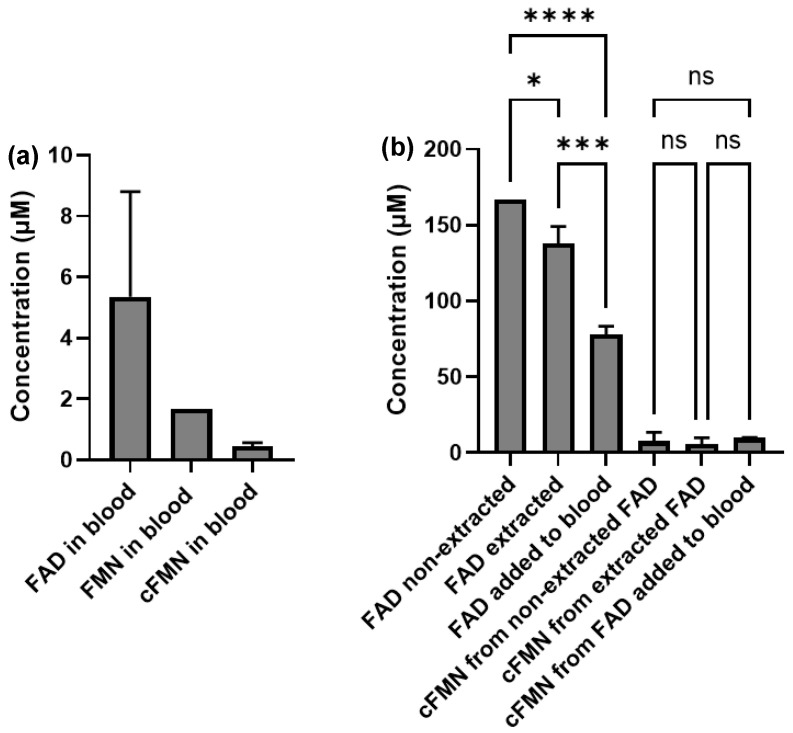
(**a**) Concentration of cFMN and FAD in blood sample extract measured with LC-MS. (**b**) Standard FAD analyzed without extraction (FAD non-extracted); standard FAD spike in water and analyzed after extraction (FAD extracted); standard FAD spiked into the blood followed by extraction (FAD added to blood). Statistical analysis using two-way ANOVA. ns *p* > 0.05; * *p* ≤ 0.05; *** *p* ≤ 0.001; **** *p* ≤ 0.0001 *n* = 3 technical replicates and *n* = 2 biological replicates.

To establish whether LC-MS would be suitable to detect and quantify free FMN, cFMN, and FAD in blood samples, we first sought to demonstrate that endogenous cFMN and FMN could be differentiated from the cFMN and FMN putatively generated during the sample processing. Thus, we performed our standard extraction protocol for each sample using −80 °C degassed methanol, chloroform, and water. The same extraction protocol was also applied to 5 nmol aqueous solutions of FAD and cFMN, respectively. We analyzed each extracted sample, along with that of a pure solution of FAD and cFMN. FAD, FMN, and cFMN were detected in the non-spiked blood sample (Figure S20a–c) and were quantified following normalization to an external standard curve (Figure 7a).

We found that the FAD abundance in the extracted vs. non-extracted pure FAD samples was very similar (Figure S21a,d and Figure 7b) and showed no significant difference with the FAD detected in the extracted blood sample spiked with FAD (Figure S20d,c and Figure 7b). While we observed a higher abundance of FAD in the blood sample spiked with FAD compared with the non-spiked sample (Figure S20d), we also observed the highest abundance of FMN and cFMN (Figure S20e,f). Notably, the cFMN peak (not in-source generated) was observed in all samples (Figure S20a–f). As for FAD, we observed that the abundance of cFMN measured in extracted FAD-spiked blood, extracted FAD (no blood), and non-extracted FAD samples was also similar (Figure 7b). This suggested that commercial FAD contained some cFMN. This was confirmed by ^31^P-NMR (Figure S22). Although the formation of cFMN from FAD during the extraction of biospecimens may occur, we are confident that the cFMN observed in blood extracts is an endogenous species.

### 3.4. cFMN Stability to Acidic and Basic pH and Hydrolysis of cFMN by Mouse Tissue Extracts

cFMN is a five-membered ring cyclophosphate and, as such, could possess properties reminiscent of the 2′,3′-cyclonucleotides such as 2′,3′-cAMP and 2′,3′-cGMP, or possibly of the 3′,5′-cyclonucleotides such as 3′,5′-cAMP, although the latter is a six-membered ring cyclic phosphate. Like its nucleotide counterparts, we found cFMN stable in acidic and basic conditions (Figures S23–S25). Studies showed that light can dealkylate riboflavin to form lumiflavin or lumichrome and causes the further decomposition of the isoalloxazine ring [25]. As such, cFMN can be stored at room temperature (rt ~20 °C) in solution or as a solid for weeks when kept away from direct light.

**Figure 8:**
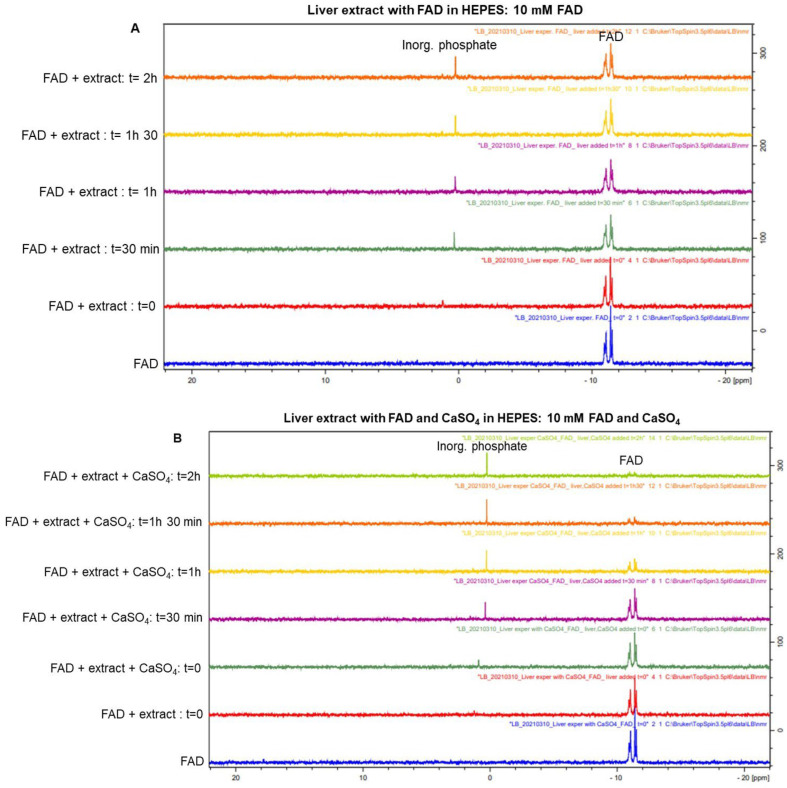

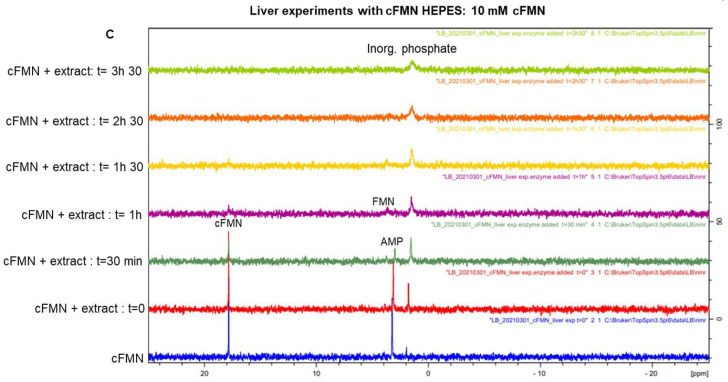
Hydrolysis of cFMN (10 mM) and FAD (10 mM) by mouse liver extracts. (**A**) ^31^P NMR of the slow hydrolysis of FAD to inorganic phosphate; (**B**) ^31^P NMR of the fast hydrolysis of FAD to inorganic phosphate enabled by calcium; (**C**) ^31^P NMR of the fast hydrolysis of cFMN and AMP to inorganic phosphate.

Using ^31^P-NMR, we then examined whether cFMN was biochemically converted to FMN or riboflavin and found that the conversion of FAD and cFMN to riboflavin was facilitated by mouse liver extract and divalent cations, like Ca^2+^ (Figure 8A–C). In addition to observing the loss of cFMN and its conversion to FMN, we also observed a broadening of the peaks in the phosphate region (0–4 ppm). We also detected the loss of the isoalloxazine hydrogen signals by ^1^H NMR (7–9 ppm). This loss was not associated with the appearance of new tricyclic signals corresponding to riboflavin degradation products but instead indicated the loss of “in-solution riboflavin”. Riboflavin has a high affinity for globulin [3]. The liver extracts used for this set of experiments are likely sources of binding proteins that bind riboflavin in solution.

**Figure 9:**
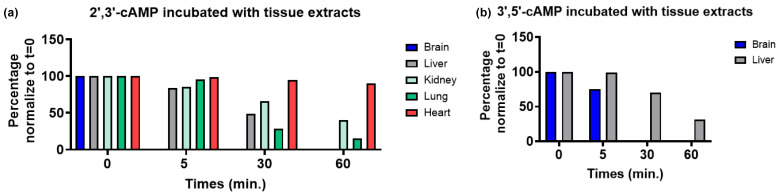
Phosphodiesterases-rich protein extracts (2.5 mg/mL) of mouse tissues that hydrolyze: (**a**) 2’,3’-cAMP (2 mM); (**b**) 3’,5’-cAMP (2 mM). The reactions were monitored by ^31^P-NMR (ns = 40).

**Figure 10:**
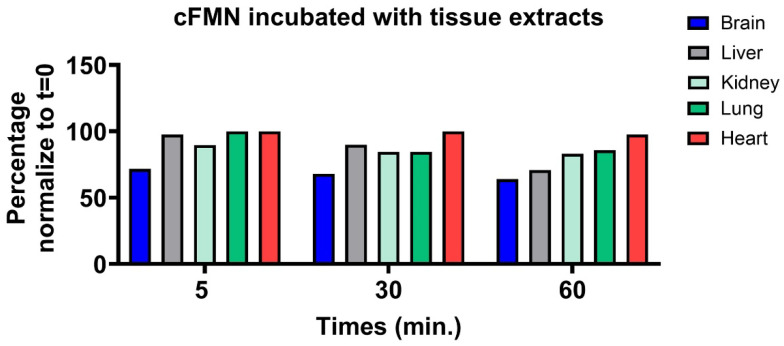
Phosphodiesterases-rich protein extracts (2.5 mg/mL) of mouse tissue that hydrolyze cFMN (2 mM). The reactions were monitored by ^31^P-NMR (ns = 40).

The hydrolytic cleavage of cFMN to FMN is a key step in the degradation of FAD to riboflavin and phosphate. Several enzymes are known to catalyze the hydrolysis of cyclic nucleotides [41]. Among them, CNPase cleaves 2′,3′-cyclic nucleotides to nucleotides, as occurs in the transformation of 2′,3′-cyclic phospho-NAD (cNADP) into NADP^+^ [42]. This hydrolysis is fast and can be monitored by LC-MS (Figure S26). Attempts to hydrolyze cFMN with CNPase proved unsuccessful (Figure S27). Using ^31^P-NMR to monitor the reactions, we then evaluated whether tissues containing high levels of phosphodiesterases (PDEs) could hydrolyze cFMN since these enzymes are known to hydrolyze 3′, 5′-cyclic nucleotides like cAMP and cGMP [43]. Although we observed the hydrolysis of cFMN, the rates of conversion to FMN and, ultimately, to inorganic phosphate were sluggish compared to cAMP hydrolysis (Figure 9, Figure 10 and Figure S28–S39). It is apparent that while liver extract readily hydrolyzes cFMN to FMN and riboflavin, enzymes known to hydrolyze canonical cyclic nucleotides to nucleosides are not optimal catalysts for cFMN hydrolysis under the conditions used in this assay, and more investigations are warranted.

## 4. Discussion

Riboflavin, FAD, and FMN are chemically unstable because they readily react with light, oxygen, and other oxidizing agents [25]. Yet, they are central cofactors to biochemical homeostasis [35,44]. Although our understanding of the biosynthetic pathway that maintains intracellular FMN and FAD levels from an exogenous riboflavin source is growing, the concentration of free intracellular FAD might be under-evaluated, and that of cFMN entirely ignored, given the facile degradation of FAD and the difficulty in differentiating and quantifying FAD, FMN, and cFMN by spectroscopic methods. Using synthetic cFMN as an external standard for LC-MS, we can now quantify cFMN present in biospecimen extracts. The observation that cFMN is metabolized by liver extracts supports the possibility that cFMN is an endogenous FAD metabolite that requires degradation. Overall, the fate of FAD could be more complex than anticipated, as cFMN could be a yet-to-be-explored riboflavin metabolite.

Current methods do not measure cFMN or cannot differentiate cFMN from FMN (i.e., fluorescence), although this cleavage, revealed exquisitely by ^31^P-NMR, proves to occur readily. In a publication from 1980, the authors observed that plasma (rich in divalent cations, e.g., [Ca^2+^] 2.2–2.6 mM) led to the rapid hydrolysis of FAD to FMN, while the conversion of FAD to FMN was slow when catalyzed by erythrocytes (low free [Ca^2+^]) [45]. We hypothesize that this observation was a direct consequence of cFMN formation from FAD, and that cFMN was the major product detected when serum was applied to FAD. Since FMN and cFMN cannot be differentiated by standard fluorescence-based analyses, the predominance of one pathway over another has not been established under physiologically relevant conditions.

The steric freedom of the C4’-hydroxyl of the riboflavin moiety is likely responsible for the ease with which FAD is converted to cFMN in the presence of cations. Based on this premise, the rapid conversion of FAD to cFMN enabled by TKFC might be a coincidence enabled by Ca^2+^ in TKFC’s ATP binding pocket. Since TKFC binds the adenosine diphosphate unit of FAD in a complex with Ca^2+^, the hydroxyl at the C4’-position of the flavin-ribotide acts as the most flexible nucleophilic alcohol that mimics a nucleophilic triose. This might minimize nonproductive binding of adenosine diphosphate species like FAD in the TKFC binding pocket. Notably, this off-target behavior might also be observed for ATP hydrolases and transferases that are Ca^2+^- or Zn^2+^-dependent and recognize FAD.

Evidently, cations affect the stability of the pyrophosphate bond in FAD. On the one hand, the cation-enabled mechanism revealed by this work raises the possibility that freed intracellular Ca^2+^ or Zn^2+^ during sample processing and/or storage of biospecimens are responsible for cFMN formation. On the other hand, it is possible that in FAD-rich organelles, uncontrolled fluxes of such divalent cations facilitate the formation of cFMN from FAD and FAD-bound proteins, with yet-unknown physiological consequences. Considering the ease of FAD cyclization and cFMN formation upon exposure to Ca^2+^ and Zn^2+^, the fact that these two cations are central to cellular homeostasis but detrimental to cell survival upon release in the intracellular environment is interesting [46]. In the cytosol, Mg^2+^ is present in millimolar concentrations while calcium and zinc are present only in nanomolar concentrations. Although the cytosol is where FMN and FAD are synthesized from riboflavin, FAD/FMN-dependent cellular processes are more prominent in other organelles, such as the endoplasmic reticulum, the peroxisome, and the mitochondrion. Indeed, FAD levels are crucial to the endoplasmic reticulum (ER), but unlike the cytosol, the ER serves as cellular calcium storage and reaches high, micro- to millimolar calcium levels. The induction of Ca^2+^ from its storage could activate cFMN formation and FAD degradation in this organelle. The capacity of enzymes to generate cFMN, serendipitously or purposefully, could be a yet-to-be-investigated cell-death-promoting mechanism when enzyme flux exceeds homeostatic conditions. Alternatively, there is also the possibility that cFMN has some downstream function(s) in response to fluctuating cation levels and Ca^2+^ signaling or acting as a signaling molecule with properties similar to those of cyclic-nucleic acids (e.g., 2′, 3′-cNMP).

## 5. Conclusions

Here, we report that FAD decomposition to AMP and cFMN is greatly enabled by divalent cations, such as Ca^2+^ and Zn^2+^, at physiologically relevant pH levels, and that Zn^2+^ is most effective at generating clean cFMN from FAD by precipitating AMP as a zinc salt. We also report that although cFMN is very stable in response to pH changes, it is slowly degraded to FMN and ultimately to riboflavin in the presence of liver extracts. We conclude that cFMN is readily formed under non-enzymatic conditions, is detectable in tissues, and can be metabolized to riboflavin by yet-unknown enzymatic processes. Furthermore, in this work, we claim that FAD conversion to cFMN is more common than expected and that this conversion goes unnoticed, as it is prone to occur under conditions that contain divalent cations. There is a possibility that functional FMN levels in cells and tissues are not adequately quantified in certain pathologies since cFMN cannot directly form FAD and can be readily mistaken for FMN under fluorometric analyses. Although LC-MS provides a solution, measurements of endogenous FAD, FMN, and cFMN will require internal, isotope-labeled standards before they can be used to inform us about the effects of sample handling on the endogenous FAD, FMN, and cFMN pools during sample processing, given FAD’s reactivity towards degradation to cFMN. Finally, to establish whether the FAD to FMN to riboflavin metabolic pathway is more prevalent than the FAD to cFMN to FMN to riboflavin pathway under physiological conditions, ^31^P-NMR (and ultimately ^31^P-MRS) or LC-MS analytical methods will need to be further optimized.

## Figures and Tables

**Table 1:** Reaction outcomes between FAD (10 mM) and metal cations (10 mM) under variable pH, temperatures, and buffer conditions. Cl^−^ (20 mM) and SO_4_^2−^ (10 mM) are released from MgCl_2_, ZnSO_4,_ and CaSO_4_, respectively. * % Conversion measured by ^31^P-NMR and ^1^H-NMR in 50 mM buffer (addition of 10% D_2_O at the time of the measurement and normalized to trimethyl phosphate as internal standard).

Buffers	Times (h)	Temp.	pH	Cations	% Conversion to cFMN and AMP *
Tris-HCl	24	37 °C	7.4	Mg^++^	24
				Ca^++^	99
				Zn^++^	82
			8	Mg^++^	42
				Ca^++^	96
				Zn^++^	94
		50 °C	7.4	Mg^++^	83
				Ca^++^	95
				Zn^++^	97
			8	Mg^++^	90
				Ca^++^	97
				Zn^++^	92
HEPES	24	50 °C	7.4	Mg^++^	82
				Ca^++^	99
				Zn^++^	95
	3	37 °C	8	Mg^++^	42
				Ca^++^	87
				Zn^++^	94
	6			Mg^++^	57
				Ca^++^	94
				Zn^++^	98
PBS	3	37 °C	7.4	Mg^++^	10
				Ca^++^	9
				Zn^++^	24
	6			Mg^++^	26
				Ca^++^	11
				Zn^++^	41
	3		8	Mg^++^	9
				Ca^++^	6
				Zn^++^	9
	6			Mg^++^	20
				Ca^++^	7
				Zn^++^	15

## Data Availability

The original contributions presented in this study are included in the article/Supplementary Materials. Further inquiries can be directed to the corresponding authors.

## Supplementary Materials

The following supporting information can be downloaded at: https://www.mdpi.com/article/10.3390/biom16010175/s1. Experimental NMR and LC-MS spectra are provided in the supplementary files. Scheme S1: Respective pathways of FAD degradation to riboflavin and the relevance of cFMN formation in the context of divalent cation physiology. Figure S1. ^1^H NMR (10% D_2_O in H_2_O, 400 MHz, T = 25 °C) of FAD. Figure S2. ^31^P NMR (10% D_2_O in H_2_O, 400 MHz, T = 25 °C) of FAD. Figure S3. ^1^H NMR (10% D_2_O in H_2_O, 400 MHz, T = 25 °C) of FAD. Figure S4. ^31^P NMR (10% D_2_O in H_2_O, 400 MHz, T = 25 °C) of FAD. Figure S5. ^1^H NMR (10% D_2_O in H_2_O, 400 MHz, T = 25 °C) of FAD. Figure S6. ^31^P NMR (10% D_2_O in H_2_O, 400 MHz, T = 25 °C) of FAD. Figure S7. ^1^H NMR (10% D_2_O in H_2_O, 400 MHz, T = 25 °C) of the reactions of FAD. Figure S8. ^31^P NMR (10% D_2_O in H_2_O, 161.1 MHz, T = 25 °C) of the reactions of FAD. Figure S9. ^31^P NMR (10% D_2_O in H_2_O, 161.1 MHz, T = 25 °C) of the reactions of FAD (10 mM) to form cFMN in Tris-HCl (50 mM) as a function of divalent cations (10 mM) at pH 7.4 and 50 °C in 24 h. (a) ZnSO_4_; (b) CaSO_4_; (c) MgCl_2_.Figure S10. ^31^P NMR (10% D_2_O in H_2_O, 161.1 MHz, T = 25 °C) of the reactions of FAD (10 mM) to form cFMN in HEPES (50 mM) as a function of divalent cations (10 mM) at pH 7.4 and 50 °C in 24 h. (a) ZnSO_4_; (b) CaSO_4_; (c) MgCl_2_. Figure S11. ^31^P NMR (10% D_2_O in H_2_O, 161.1 MHz, T = 25 °C) of the reactions of FAD (10 mM) to form cFMN in HEPES (50 mM) as a function of divalent cations (10 mM) at pH 8 and 37 °C in 3 h. (a) ZnSO_4_; (b) CaSO_4_; (c) MgCl_2_.Figure S12. ^31^P NMR (10% D_2_O in H_2_O, 161.1 MHz, T = 25 °C) of the reactions of FAD (10 mM) to form cFMN in HEPES (50 mM) as a function of divalent cations (10 mM) at pH 8 and 37 °C in 6 h. (a) ZnSO_4_; (b) CaSO_4_; (c) MgCl_2._ Figure S13. ^31^P NMR (10% D_2_O in H_2_O, 161.1 MHz, T = 25 °C) of the reactions of FAD (10 mM) to form cFMN in PBS (50 mM) as a function of divalent cations (10 mM) at pH 7.4 and 37 °C in 3 h. (a) ZnSO_4_; (b) CaSO_4_; (c) MgCl_2_. Figure S14. ^31^P NMR (10% D_2_O in H_2_O, 161.1 MHz, T = 25 °C) of the reactions of FAD (10 mM) to form cFMN in PBS (50 mM) as a function of divalent cations (10 mM) at pH 7.4 and 37 °C in 6 h. (a) ZnSO_4_; (b) CaSO_4_; (c) MgCl_2_. Figure S15. ^31^P NMR (10% D_2_O in H_2_O, 161.1 MHz, T = 25 °C) of the reactions of FAD (10 mM) to form cFMN in PBS (50 mM) as a function of divalent cations (10 mM) at pH 8 and 37 °C in 3 h. (a) ZnSO_4_; (b) CaSO_4_; (c) MgCl_2_. Figure S16. ^31^P NMR (10% D_2_O in H_2_O, 161.1 MHz, T = 25 °C) of the reactions of FAD (10 mM) to form cFMN in PBS (50 mM) as a function of divalent cations (10 mM) at pH 8 and 37 °C in 6 h. (a) ZnSO_4_; (b) CaSO_4_; (c) MgCl_2_. Figure S17. Reactions of FAD (10 mM) in Tris-HCl (50 mM, pH 7.4, 37 °C) to form cFMN in the presence of different concentrations of CaSO_4_ in 24 h. Figure S18. ^31^P NMR (10% D_2_O in H_2_O, 161.1 MHz, T = 25 °C) of the reactions of FAD (10 mM) in Tris-HCl (50 mM, pH 7.4, 37 °C) to form cFMN in the presence of different concentrations of CaSO_4_ in 24 h. Figure S19. ^31^P NMR (10% D_2_O in H_2_O, 161.1 MHz, T = 25 °C) of the reactions of FAD (10 mM) in Tris-HCl (50 mM, pH 7.4, 37 °C) to form cFMN in the presence of different concentrations of CaSO_4_ in 24 h. Figure S20. HILIC LC-MS chromatograms of the measurements. Figure S21. HILIC LC-MS chromatograms of the measurements. Figure S22. 31P NMR (10% D_2_O in H_2_O, 161.1 MHz, T = 25 °C) of FAD showing cFMN contamination at 17.9 ppm. Figure S23. ^31^P NMR (10% D_2_O in H_2_O, 161.1 MHz, T = 25 °C) of cFMN and AMP incubated in water at pH 4 and 37 °C. Figure S24. ^31^P NMR (10% D_2_O in H_2_O, 161.1 MHz, T = 25 °C) of cFMN and AMP incubated in water at pH 7 and 37 °C. Figure S25. ^31^P NMR (10% D_2_O in H_2_O, 161.1 MHz, T = 25 °C) of cFMN and AMP incubated in water at pH 9.2 and 37 °C. Figure S26. Mass Spectrometry chromatograms of the reaction of cNADP^+^ by CNPase to form NADP^+^. Figure S27. Mass Spectrometry chromatograms of the reaction of cFMN by CNPase to form FMN. Figure S28. Brain-rich protein extracts (2.5 mg/mL) of mice tissue that hydrolyze 2′,3′-cAMP (2 mM). Figure S29. Liver-rich protein extracts (2.5 mg/mL) of mice tissue that hydrolyze 2′,3′-cAMP (2 mM). Figure S30. Kidney-rich protein extracts (2.5 mg/mL) of mice tissue that hydrolyze 2′,3′-cAMP (2 mM). Figure S31. Lung-rich protein extracts (2.5 mg/mL) of mice tissue that hydrolyze 2′,3′-cAMP (2 mM). Figure S32. Heart-rich protein extracts (2.5 mg/mL) of mice tissue that hydrolyze 2′,3′-cAMP (2 mM). Figure S33. Brain-rich protein extracts (2.5 mg/mL) of mice tissue that hydrolyze 3′,5′-cAMP (2 mM). Figure S34. Liver-rich protein extracts (2.5 mg/mL) of mice tissue that hydrolyze 3′,5′-cAMP (2 mM). Figure S35. Brain-rich protein extracts (2.5 mg/mL) of mice tissue that hydrolyze cFMN (2 mM). Figure S36. Liver-rich protein extracts (2.5 mg/mL) of mice tissue that hydrolyze cFMN (2 mM). Figure S37. Kidney-rich protein extracts (2.5 mg/mL) of mice tissue that hydrolyze cFMN (2 mM). Figure S38. Lung-rich protein extracts (2.5 mg/mL) of mice tissue that hydrolyze cFMN (2 mM). Figure S39. Heart-rich protein extracts (2.5 mg/mL) of mice tissue that hydrolyze cFMN (2 mM).

## References

[1] Thakur K., Tomar S.K., Singh A.K., Mandal S., Arora S. (2017). Riboflavin and health: A review of recent human research. Crit. Rev. Food Sci. Nutr..

[2] Balasubramaniam S., Yaplito-Lee J. (2020). Riboflavin metabolism: Role in mitochondrial function. J. Transl. Genet. Genom..

[3] Barile M. (2021). Flavins and Flavoproteins: Methods and Protocols.

[4] Suwannasom N., Kao I., Pruss A., Georgieva R., Baumler H. (2020). Riboflavin: The Health Benefits of a Forgotten Natural Vitamin. Int. J. Mol. Sci..

[5] Alam M.M., Iqbal S., Naseem I. (2015). Ameliorative effect of riboflavin on hyperglycemia, oxidative stress and DNA damage in type-2 diabetic mice: Mechanistic and therapeutic strategies. Arch. Biochem. Biophys..

[6] Tao Z., Huo J., Hao X., Liang J. (2025). Riboflavin in neurological diseases: Therapeutic advances, metabolic insights, and emerging genetic strategies. Front. Neurol..

[7] Garg M., Kulkarni S.D., Hegde A.U., Shah K.N. (2018). Riboflavin Treatment in Genetically Proven Brown-Vialetto-Van Laere Syndrome. J. Pediatr. Neurosci..

[8] Balasubramaniam S., Christodoulou J., Rahman S. (2019). Disorders of riboflavin metabolism. J. Inherit. Metab. Dis..

[9] Cabezas A., Costas M.J., Pinto R.M., Couto A., Cameselle J.C. (2005). Identification of human and rat FAD-AMP lyase (cyclic FMN forming) as ATP-dependent dihydroxyacetone kinases. Biochem. Biophys. Res. Commun..

[10] Lienhart W.D., Gudipati V., Macheroux P. (2013). The human flavoproteome. Arch. Biochem. Biophys..

[11] Giancaspero T.A., Busco G., Panebianco C., Carmone C., Miccolis A., Liuzzi G.M., Colella M., Barile M. (2013). FAD synthesis and degradation in the nucleus create a local flavin cofactor pool. J. Biol. Chem..

[12] Pallotta M.L., Brizio C., Fratianni A., De Virgilio C., Barile M., Passarella S. (1998). Saccharomyces cerevisiae mitochondria can synthesise FMN and FAD from externally added riboflavin and export them to the extramitochondrial phase. FEBS Lett..

[13] Rivero M., Boneta S., Novo N., Velazquez-Campoy A., Polo V., Medina M. (2023). Riboflavin kinase and pyridoxine 5′-phosphate oxidase complex formation envisages transient interactions for FMN cofactor delivery. Front. Mol. Biosci..

[14] Yoshino J., Baur J.A., Imai S.I. (2018). NAD^+^ Intermediates: The Biology and Therapeutic Potential of NMN and NR. Cell Metab..

[15] Ratajczak J., Joffraud M., Trammell S.A., Ras R., Canela N., Boutant M., Kulkarni S.S., Rodrigues M., Redpath P., Migaud M.E. (2016). NRK1 controls nicotinamide mononucleotide and nicotinamide riboside metabolism in mammalian cells. Nat. Commun..

[16] Bieganowski P., Brenner C. (2004). Discoveries of nicotinamide riboside as a nutrient and conserved NRK genes establish a Preiss-Handler independent route to NAD+ in fungi and humans. Cell.

[17] Zapata-Perez R., Wanders R.J.A., van Karnebeek C.D.M., Houtkooper R.H. (2021). NAD^+^ homeostasis in human health and disease. EMBO Mol. Med..

[18] Gossmann T.I., Ziegler M., Puntervoll P., de Figueiredo L.F., Schuster S., Heiland I. (2012). NAD^+^ biosynthesis and salvage--a phylogenetic perspective. FEBS J..

[19] Giancaspero T.A., Locato V., Barile M. (2013). A regulatory role of NAD redox status on flavin cofactor homeostasis in S. cerevisiae mitochondria. Oxidative Med. Cell. Longev..

[20] Bogachev A.V., Baykov A.A., Bertsova Y.V. (2018). Flavin transferase: The maturation factor of flavin-containing oxidoreductases. Biochem. Soc. Trans..

[21] Islam F., Shilton B. (2025). Insights into the cellular function and mechanism of action of quinone reductase 2 (NQO2). Biochem. J..

[22] Smith M.M., Moran G.R. (2024). Building on a theme: The redox hierarchy of pyridine nucleotide-disulfide oxidoreductases. Arch. Biochem. Biophys..

[23] Macheroux P., Kappes B., Ealick S.E. (2011). Flavogenomics—A genomic and structural view of flavin-dependent proteins. FEBS J..

[24] Marcuello C., de Miguel R., Martinez-Julvez M., Gomez-Moreno C., Lostao A. (2015). Mechanostability of the Single-Electron-Transfer Complexes of Anabaena Ferredoxin-NADP^+^ Reductase. Chemphyschem.

[25] Sheraz M.A., Kazi S.H., Ahmed S., Anwar Z., Ahmad I. (2014). Photo, thermal and chemical degradation of riboflavin. Beilstein J. Org. Chem..

[26] Lynch J.H., Sa N., Saeheng S., Raffaelli N., Roje S. (2018). Characterization of a non-nudix pyrophosphatase points to interplay between flavin and NAD(H) homeostasis in Saccharomyces cerevisiae. PLoS ONE.

[27] Pinto R.M., Fraiz F.J., Cabezas A., Avalos M., Canales J., Costas M.J., Cameselle J.C. (1999). Preparation of riboflavin 4′,5′-cyclic phosphate by incubation of flavin-adenine dinucleotide with Mn^2+^ in the absence of riboflavin 5′-phosphate cyclase. Anal. Biochem..

[28] Rodrigues J.R., Couto A., Cabezas A., Pinto R.M., Ribeiro J.M., Canales J., Costas M.J., Cameselle J.C. (2014). Bifunctional homodimeric triokinase/FMN cyclase: Contribution of protein domains to the activities of the human enzyme and molecular dynamics simulation of domain movements. J. Biol. Chem..

[29] Rodrigues J.R., Cameselle J.C., Cabezas A., Ribeiro J.M. (2019). Closure of the Human TKFC Active Site: Comparison of the Apoenzyme and the Complexes Formed with Either Triokinase or FMN Cyclase Substrates. Int. J. Mol. Sci..

[30] Rhee H.W., Choi S.J., Yoo S.H., Jang Y.O., Park H.H., Pinto R.M., Cameselle J.C., Sandoval F.J., Roje S., Han K. (2009). A bifunctional molecule as an artificial flavin mononucleotide cyclase and a chemosensor for selective fluorescent detection of flavins. J. Am. Chem. Soc..

[31] Scola-Nagelschneider G., Hemmerich P. (1976). Synthesis, separation, identification and interconversion of riboflavin phosphates and their acetyl derivatives: A reinvestigation. Eur. J. Biochem..

[32] Hayat F., Makarov M.V., Belfleur L., Migaud M.E. (2022). Synthesis of Mixed Dinucleotides by Mechanochemistry. Molecules.

[33] Jacobson R.L., Langmuir D. (1974). Dissociation constants of calcite and CaHC0_3_^+^ from 0 to 50 °C. Geochim. Cosmochim. Acta.

[34] McDonogh D.P., Gale J.D., Raiteri P., Gebauer D. (2024). Redefined ion association constants have consequences for calcium phosphate nucleation and biomineralization. Nat. Commun..

[35] Patel A., Simkulet M., Maity S., Venkatesan M., Matzavinos A., Madesh M., Alevriadou B.R. (2022). The mitochondrial Ca^2+^ uniporter channel synergizes with fluid shear stress to induce mitochondrial Ca^2+^ oscillations. Sci. Rep..

[36] Zhang B., Hou S., Tang J. (2025). Riboflavin Deficiency and Apoptosis: A Review. J. Nutr..

[37] Popoiu T.A., Maack C., Bertero E. (2023). Mitochondrial calcium signaling and redox homeostasis in cardiac health and disease. Front. Mol. Med..

[38] Hustad S., McKinley M.C., McNulty H., Schneede J., Strain J.J., Scott J.M., Ueland P.M. (2002). Riboflavin, flavin mononucleotide, and flavin adenine dinucleotide in human plasma and erythrocytes at baseline and after low-dose riboflavin supplementation. Clin. Chem..

[39] Hustad S., Ueland P.M., Schneede J. (1999). Quantification of Riboflavin, Flavin Mononucleotide, and Flavin Adenine Dinucleotide in Human Plasma by Capillary Electrophoresis and Laser-induced Fluorescence Detection. Clin. Chem..

[40] Sun K., Jiao C., Panconesi R., Satish S., Karakaya O.F., De Goeij F.H.C., Diwan T., Ali K., Cadinu L.A., Cazzaniga B. (2025). Quantifying Flavin mononucleotide: An internationally validated methodological approach for enhanced decision making in organ transplantation. eBioMedicine.

[41] Salter E.A., Wierzbicki A. (2007). The Mechanism of Cyclic Nucleotide Hydrolysis in the Phosphodiesterase Catalytic Site. J. Phys. Chem. B.

[42] Olga K., Yulia B., Vassilios P. (2020). The Functions of Mitochondrial 2′,3′-Cyclic Nucleotide-3′-Phosphodiesterase and Prospects for Its Future. Int. J. Mol. Sci..

[43] Kelly M.P., Nikolaev V.O., Gobejishvili L., Lugnier C., Hesslinger C., Nickolaus P., Kass D.A., Pereira de Vasconcelos W., Fischmeister R., Brocke S. (2025). Cyclic nucleotide phosphodiesterases as drug targets. Pharmacol. Rev..

[44] Giancaspero T.A., Colella M., Brizio C., Difonzo G., Fiorino G.M., Leone P., Brandsch R., Bonomi F., Iametti S., Barile M. (2015). Remaining challenges in cellular flavin cofactor homeostasis and flavoprotein biogenesis. Front. Chem..

[45] Okumura M., Yagi K. (1980). Hydrolysis of flavin adenine dinucleotide and flavin mononucleotide by rabbit blood. J. Nutr. Sci. Vitaminol.

[46] Di Filippo E.S., Checcaglini F., Fano-Illic G., Fulle S. (2022). H_2_O_2_/Ca^2+^/Zn^2+^ Complex Can Be Considered a “Collaborative Sensor” of the Mitochondrial Capacity?. Antioxidants.

