# Supplementary material for: Cyclic-FMN Is a Detectable, Putative Intermediate of FAD Metabolism
Source: Biomolecules. 2026 Jan 21;16(1):175. doi: 10.3390/biom16010175 (PMC12838640; doi:10.3390/biom16010175)
Supplement: Supplementary file 1 [file biomolecules-16-00175-s001.zip › biomolecules-4063813-supplementary.pdf]

Cyclic-FMN is an intermediate of FAD metabolism

Supporting information

LC-MS method for monitoring CNPase experiments

Mass UPLC-HRMS analysis has been performed using a Dionex UltiMate 3000 liquid chromatography system (UPLC) interfaced with a QExactive mass spectrometer (HRMS) equipped with an electrospray ionization source (Thermo Fischer Scientific). Analytes were separated on a ZIC-HILIC column (50x2.1mm, 100 Å, 3.5µm from Merck) kept at 50°C during the analysis. Total run time was 8 min and injection volume was 10 µL. Separation was done at flow rate of 0.3 µl/min. Solvent A and B were 3% and 90% acetonitrile respectively, both buffered with 10 mM ammonium acetate. The gradient (B) composition was: 85-80% over 1,6 min, 80-65% for the next 4,3 min, 65-40% over 0.7 min followed by 2 min of column equilibration 85%. Ions were monitored in positive targeted single ion monitoring (t-SIM) modes. t-SIM parameters are listed in table 1. Other MS parameters were automatic gain control (AGC) target of 2E5, maximum injection time 200 seconds, sheath gas flow rate 48 (arbitrary units), aux gas flow rate 11 (arbitrary units), sweep gas flow rate 2 (arbitrary units), spray voltage 3.5 kV, capillary temperature 256°C and S-lens RF level 30.

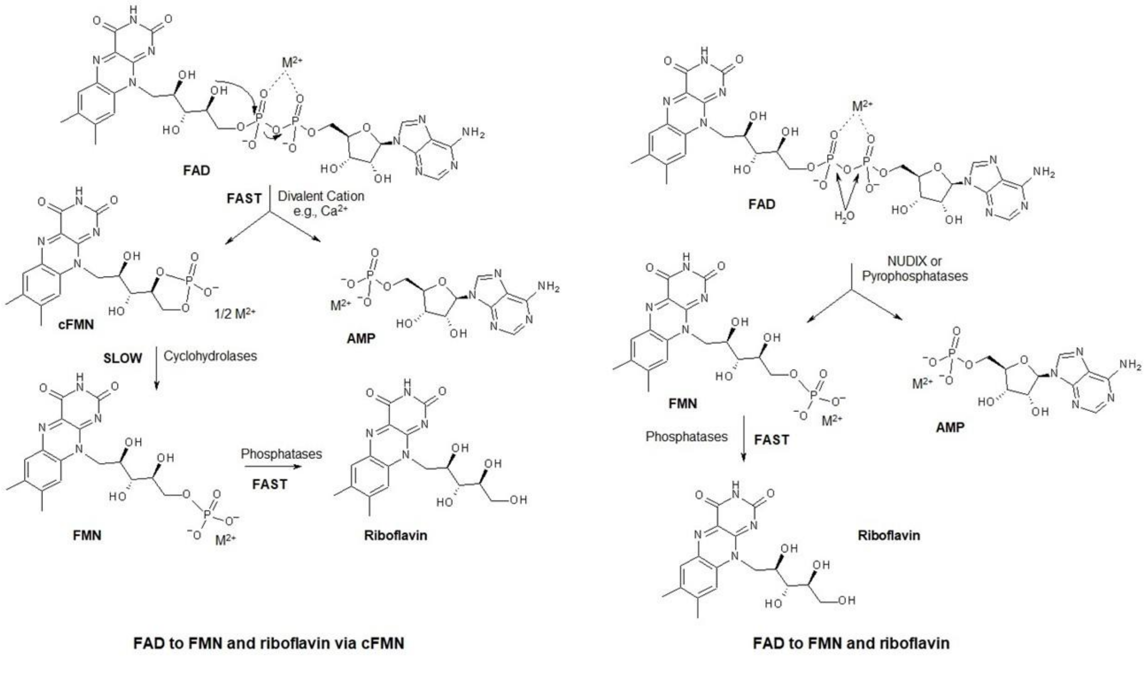

Scheme S1: Respective pathways of FAD degradation to riboflavin and the relevance of cFMN formation in the context of divalent cation physiology.

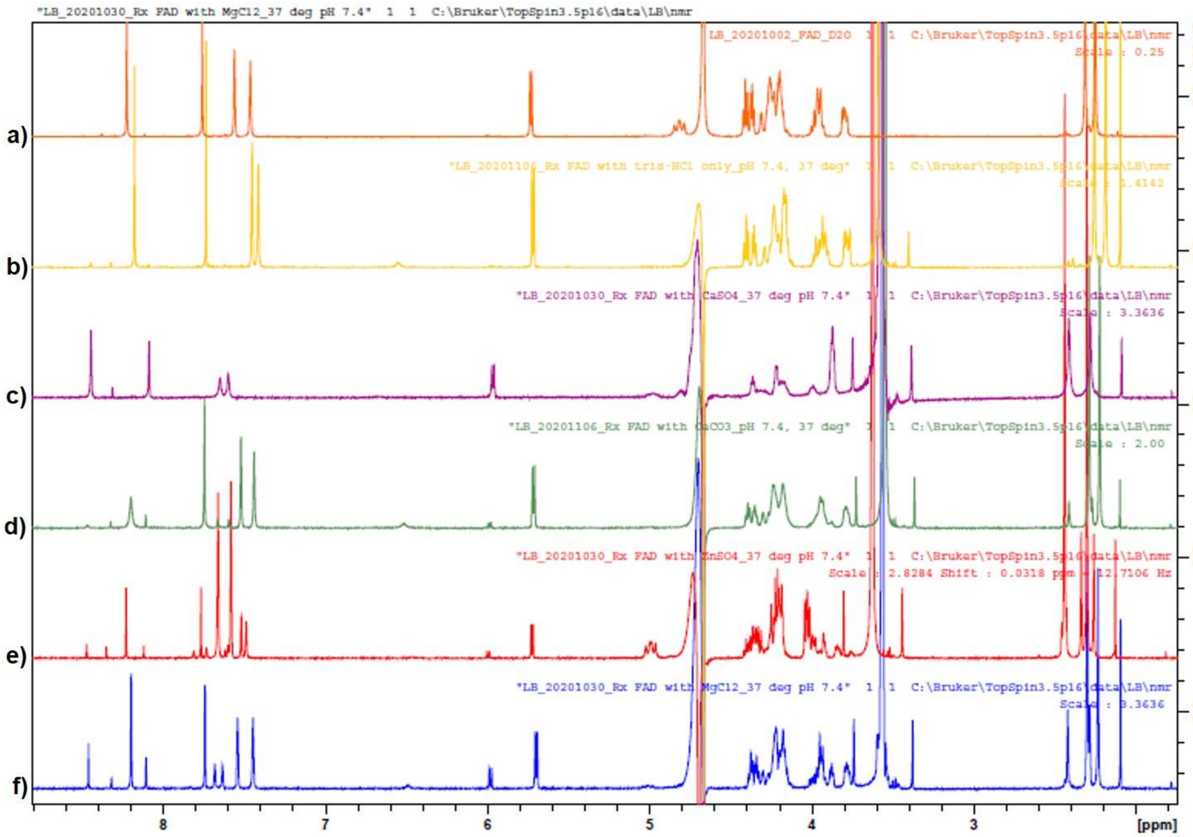

Figure S1. ^{1}H NMR (10% D_2O in H_2O, 400 MHz, T=25°C) of FAD (a); and the reactions of FAD (10 mM) with divalent cations (10 mM) to form cFMN: b) Buffer only; c) $CaSO_4$; d) $CaCO_3$; e) $ZnSO_4$; f) $MgCl_2$. The reactions are performed in Tris-HCl (50 mM) at pH 7.4 and 37 °C in 24h.

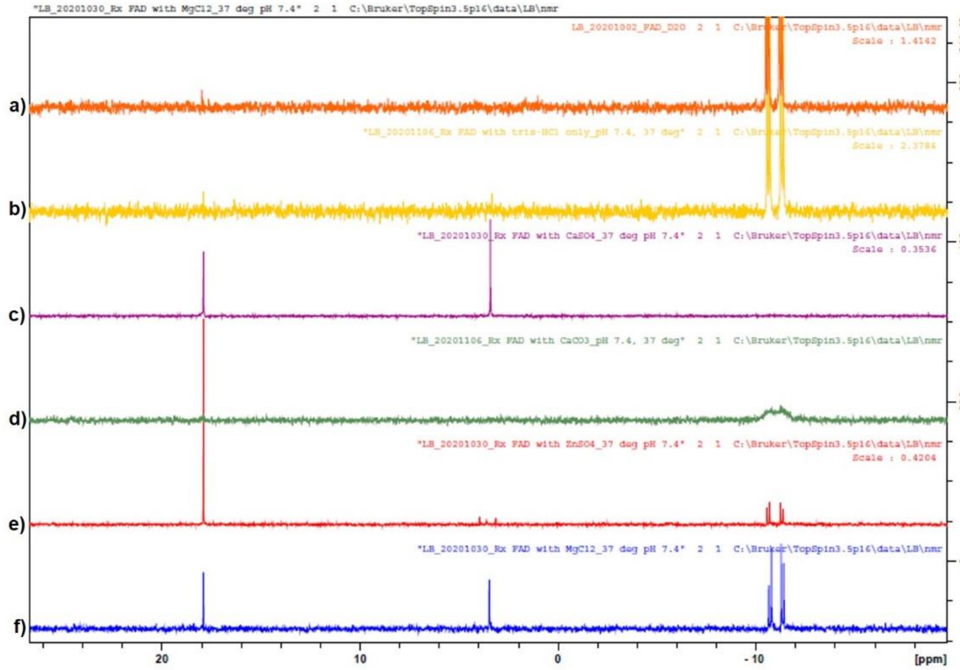

Figure S2. ^{31}P NMR (10% D_2O in H_2O, 400 MHz, T=25°C) of FAD (a); and the reactions of FAD (10 mM) with divalent cations (10 mM) to form cFMN: b) Buffer only; c) $CaSO_4$; d) $CaCO_3$; e) $ZnSO_4$; f) $MgCl_2$. The reactions are performed in Tris-HCl (50 mM) at pH 7.4 and 37 °C in 24h.

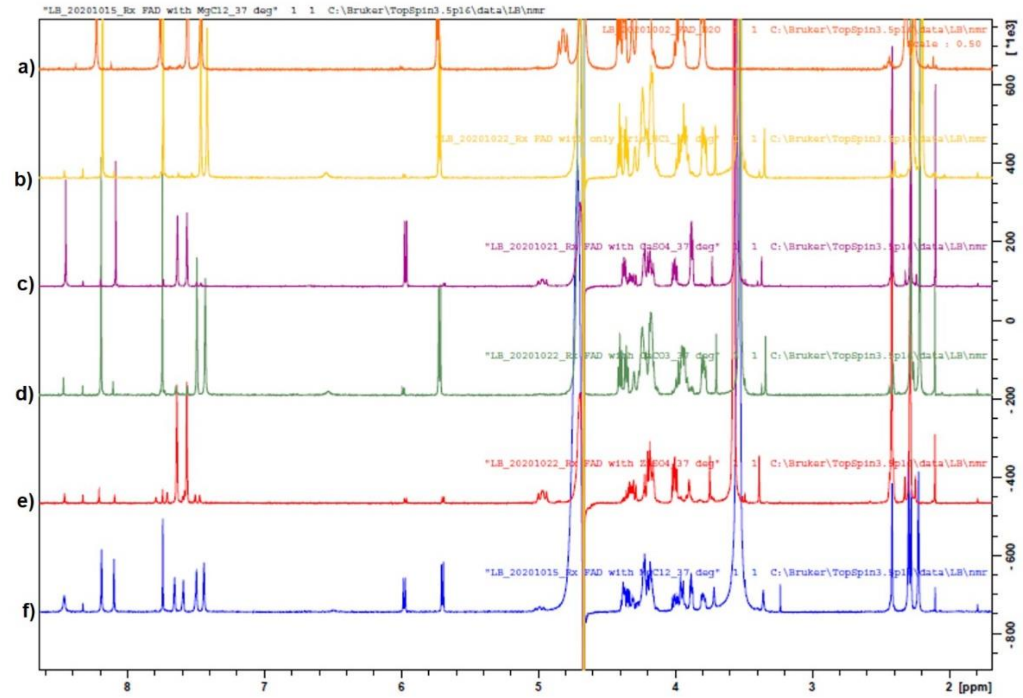

Figure S3. ^{1}H NMR (10% D_2O in H_2O, 400 MHz, T=25°C) of FAD (a); and the reactions of FAD (10 mM) with divalent cations (10 mM) to form cFMN: b) Buffer only; c) $CaSO_4$; d) $CaCO_3$; e) $ZnSO_4$; f) $MgCl_2$. The reactions are performed in Tris-HCl (50 mM) at pH 8 and 37 °C in 24h.

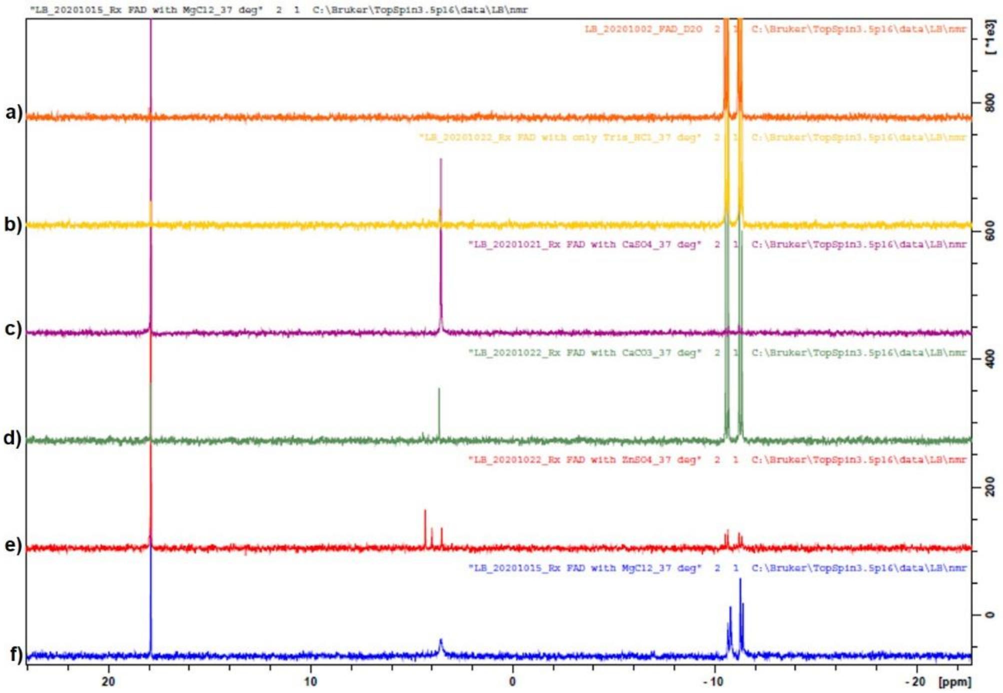

Figure S4. ^{31}P NMR (10% D_2O in H_2O, 400 MHz, T=25°C) of FAD (a); and the reactions of FAD (10 mM) with divalent cations (10 mM) to form cFMN: b) Buffer only; c) $CaSO_4$; d) $CaCO_3$; e) $ZnSO_4$; f) $MgCl_2$. The reactions are performed in Tris-HCl (50 mM) at pH 8 and 37 °C in 24h.

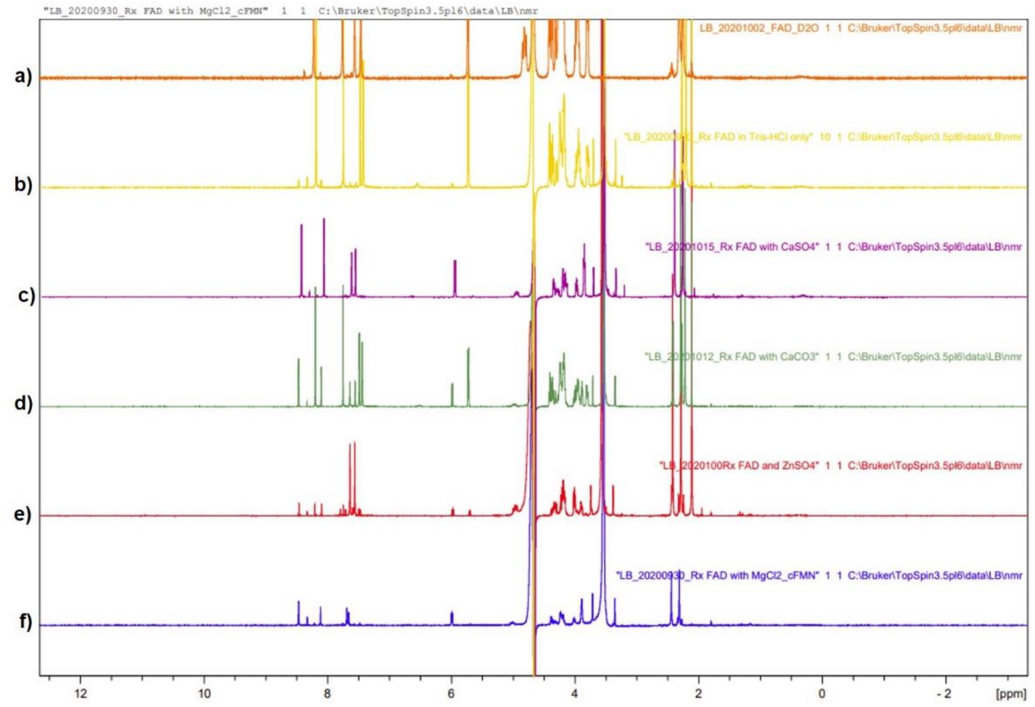

Figure S5. ^{1}H NMR (10% D_2O in H_2O, 400 MHz, T=25°C) of FAD (a); and the reactions of FAD (10 mM) with divalent cations (10 mM) to form cFMN: b) Buffer only; c) $CaSO_4$; d) $CaCO_3$; e) $ZnSO_4$; f) $MgCl_2$. The reactions are performed in Tris-HCl (50 mM) at pH 8 and 50 °C in 24h.

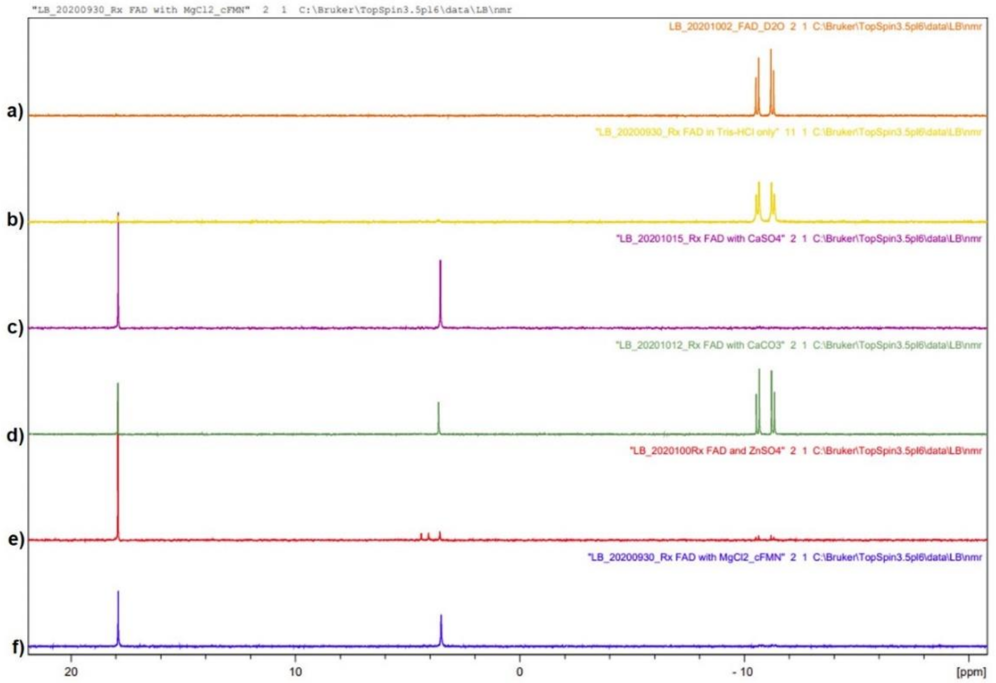

Figure S6. ^{31}P NMR (10% D_2O in H_2O, 400 MHz, T=25°C) of FAD (a); and the reactions of FAD (10 mM) with divalent cations (10 mM) to form cFMN: b) Buffer only; c) $CaSO_4$; d) $CaCO_3$; e) $ZnSO_4$; f) $MgCl_2$. The reactions are performed in Tris-HCl (50 mM) at pH 8 and 50 °C in 24h.

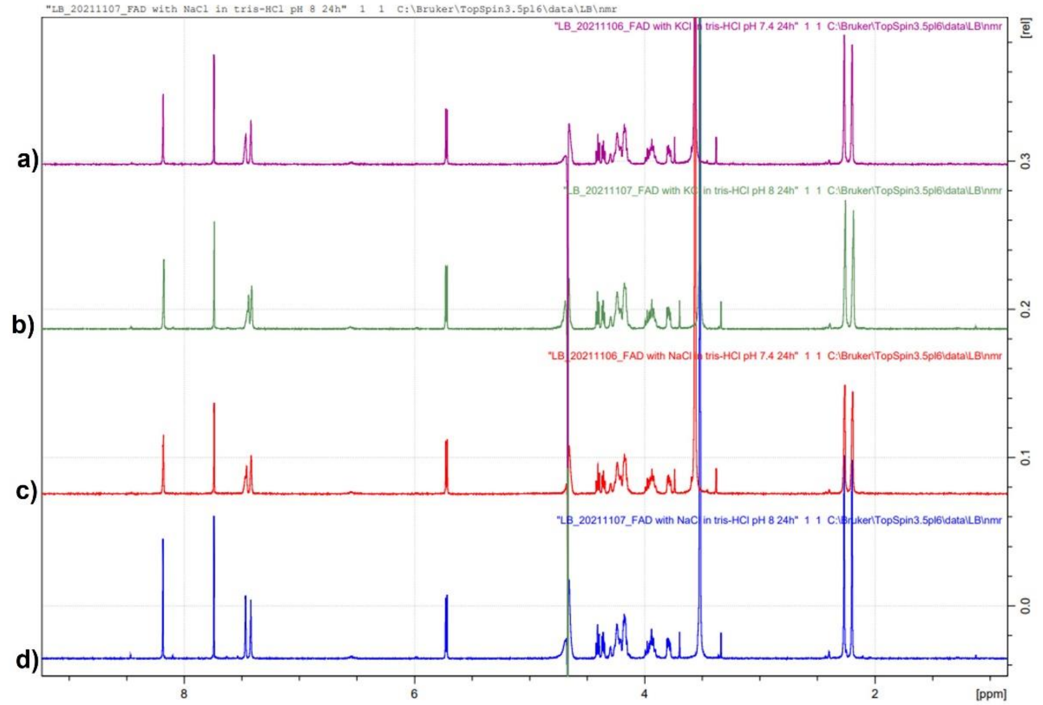

Figure S7. ^{1}H NMR (10% D_2O in H_2O, 400 MHz, T=25°C) of the reactions of FAD (10 mM) with: a) KCl (10 mM, pH 7.4); b) KCl (10 mM, pH 8); c) NaCl (10 mM, pH 7.4); d) NaCl (10 mM, pH 8). The reactions are performed in Tris-HCl (50 mM) at 37 °C in 24h.

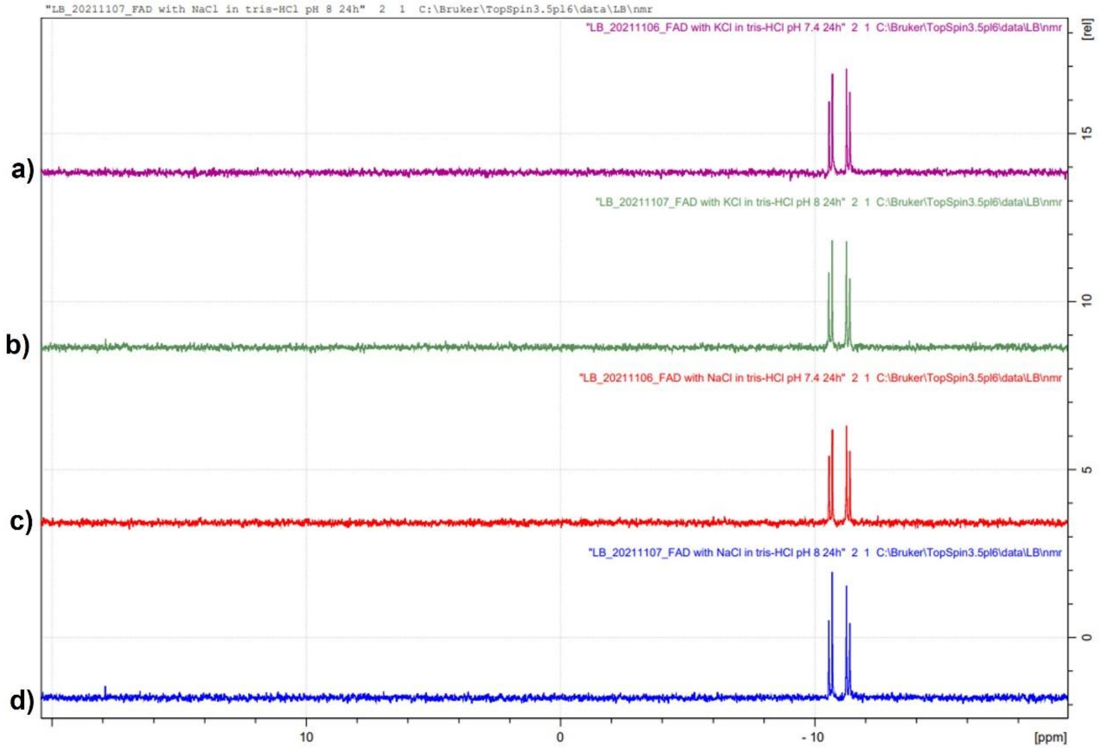

Figure S8. ^{31}P NMR (10% D_2O in H_2O, 161.1 MHz, T=25°C) of the reactions of FAD (10 mM) with: a) KCl (10 mM, pH 7.4); b) KCl (10 mM, pH 8); c) NaCl (10 mM, pH 7.4); d) NaCl (10 mM, pH 8). The reactions are performed in Tris-HCl (50 mM) at 37 °C in 24h.

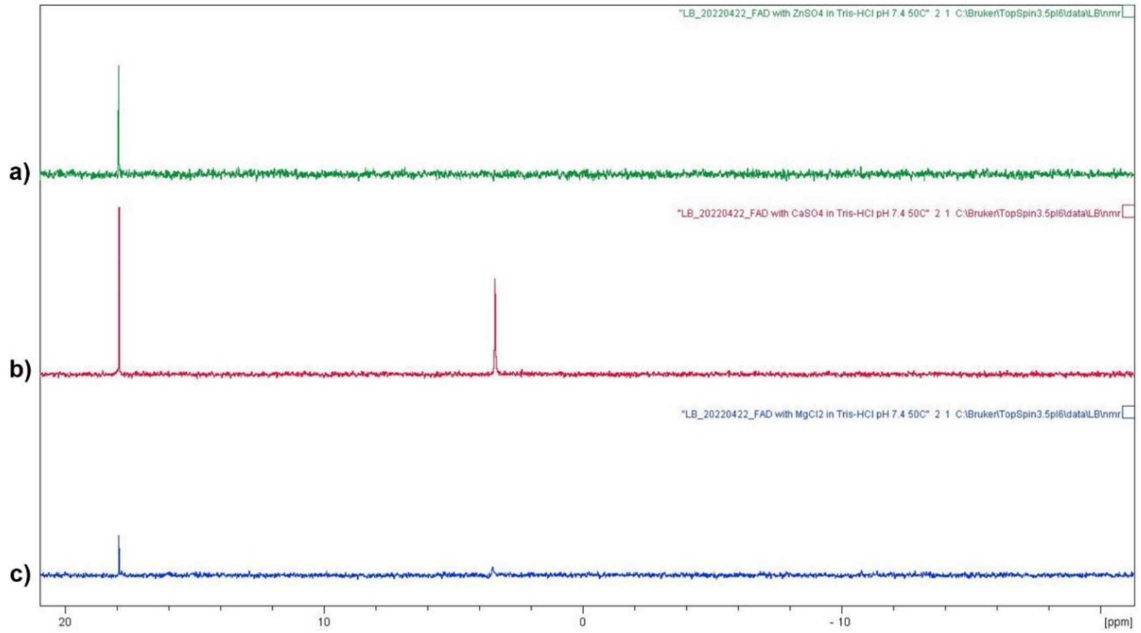

Figure S9. ^{31}P NMR (10% D_2O in H_2O, 161.1 MHz, T=25°C) of the reactions of FAD (10 mM) to form cFMN in Tris-HCl (50 mM) as a function of divalent cations (10 mM) at pH 7.4 and 50 °C in 24h. a) $ZnSO_4$; b) $CaSO_4$; c) $MgCl_2$.

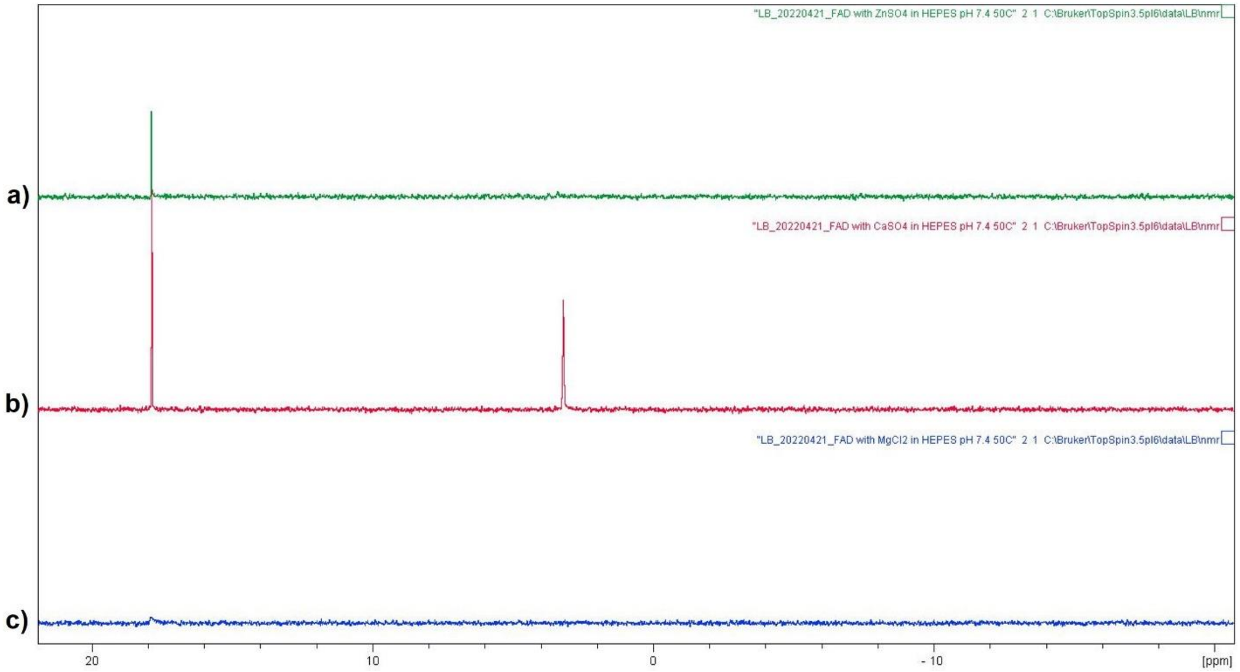

Figure S10. ^{31}P NMR (10% D_2O in H_2O, 161.1 MHz, T=25°C) of the reactions of FAD (10 mM) to

form cFMN in HEPES (50 mM) as a function of divalent cations (10 mM) at pH 7.4 and 50 °C in 24h. a) $ZnSO_4$; b) $CaSO_4$; c) $MgCl_2$.

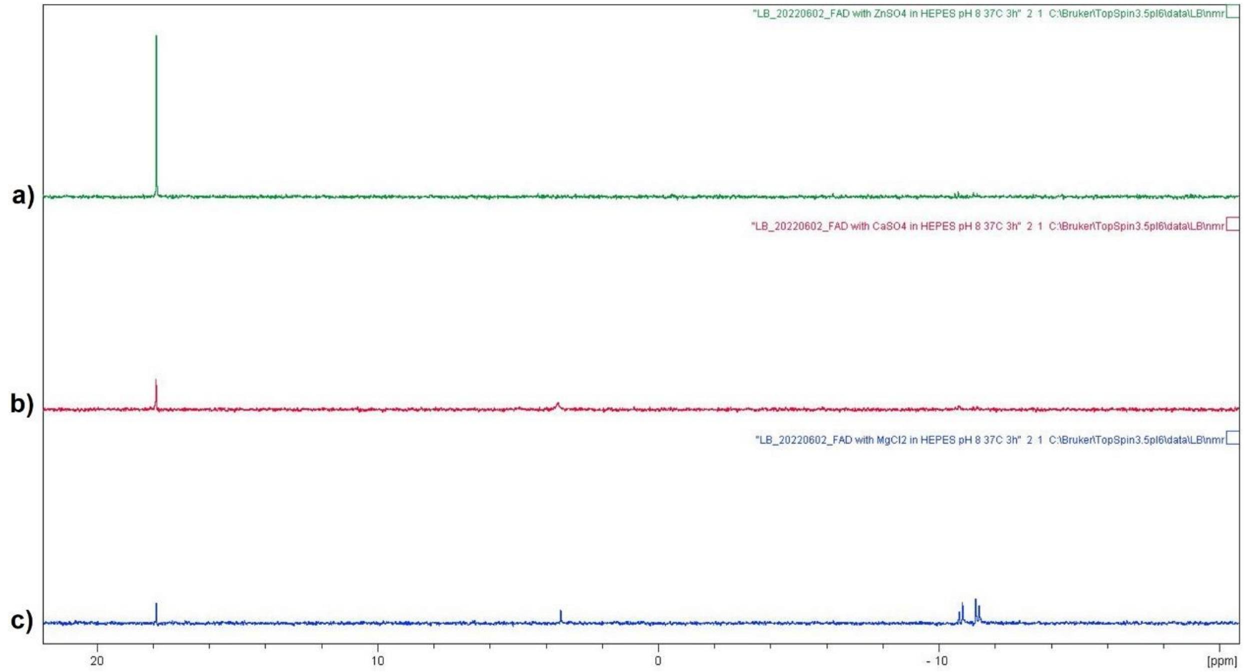

Figure S11. ^{31}P NMR (10% D_2O in H_2O, 161.1 MHz, T=25°C) of the reactions of FAD (10 mM) to form cFMN in HEPES (50 mM) as a function of divalent cations (10 mM) at pH 8 and 37 °C in 3h. a) $ZnSO_4$; b) $CaSO_4$; c) $MgCl_2$.

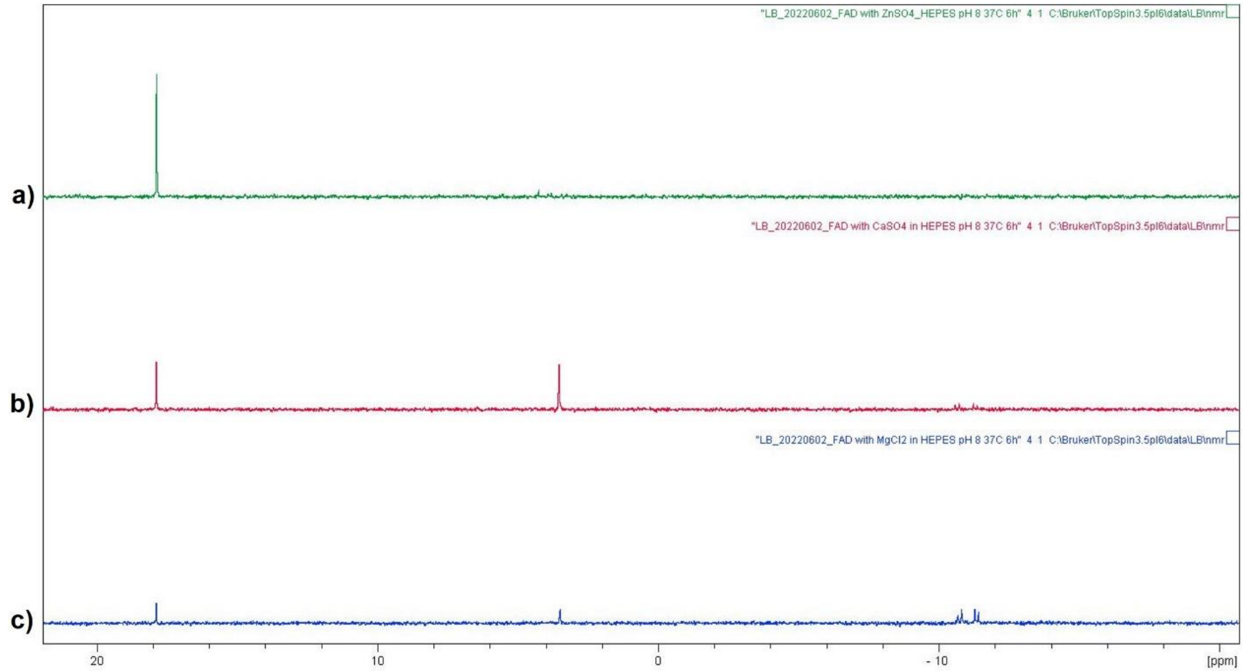

Figure S12. ^{31}P NMR (10% D_2O in H_2O, 161.1 MHz, T=25°C) of the reactions of FAD (10 mM) to form cFMN in HEPES (50 mM) as a function of divalent cations (10 mM) at pH 8 and 37 °C in 6h. a) $ZnSO_4$; b) $CaSO_4$; c) $MgCl_2$.

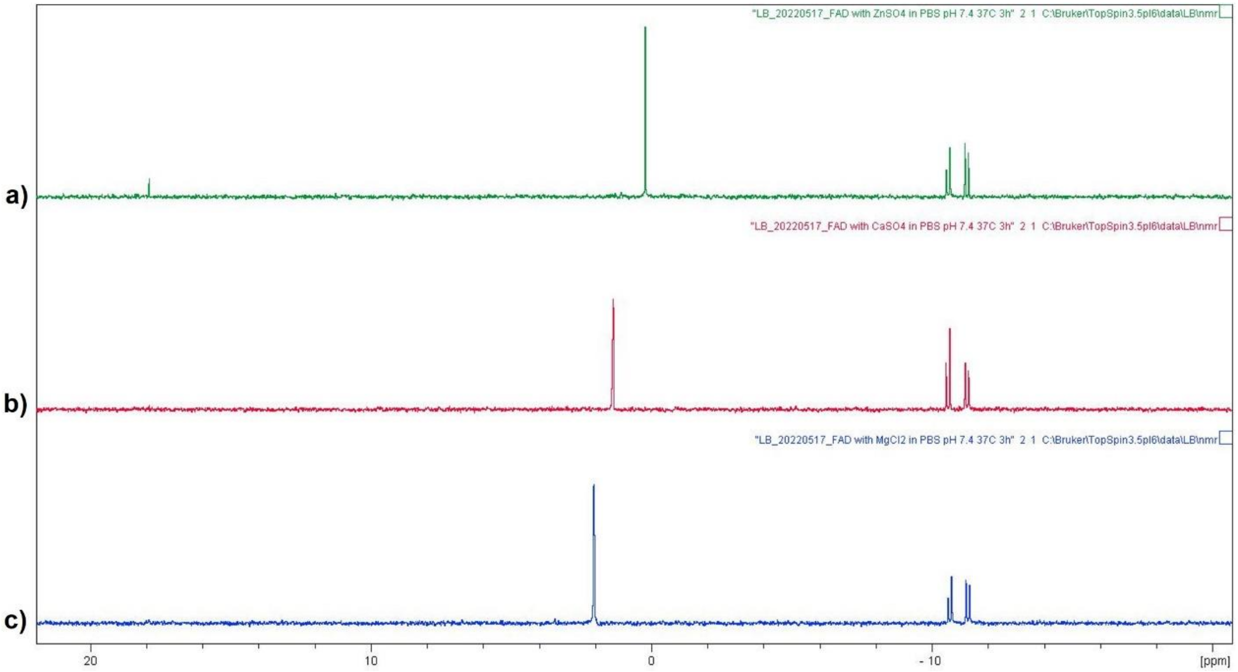

Figure S13. ^{31}P NMR (10% D_2O in H_2O, 161.1 MHz, T=25°C) of the reactions of FAD (10 mM) to form cFMN in PBS (50 mM) as a function of divalent cations (10 mM) at pH 7.4 and 37 °C in 3h. a) $ZnSO_4$; b) $CaSO_4$; c) $MgCl_2$.

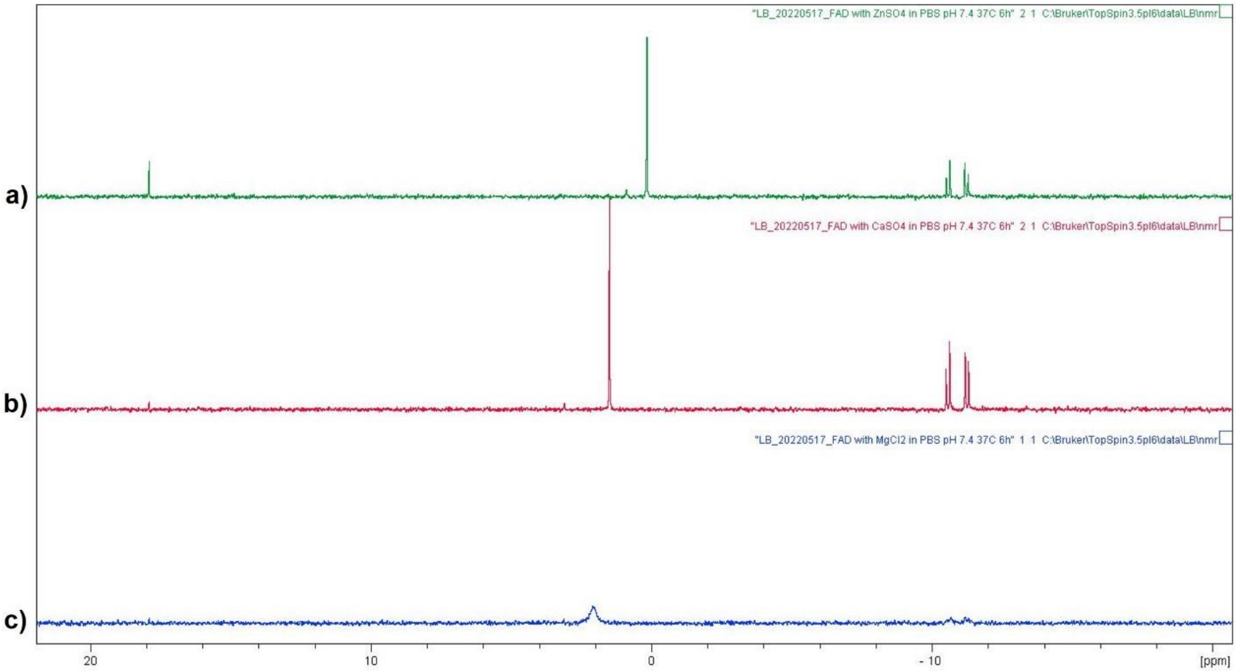

Figure S14. ^{31}P NMR (10% D_2O in H_2O, 161.1 MHz, T=25°C) of the reactions of FAD (10 mM) to form cFMN in PBS (50 mM) as a function of divalent cations (10 mM) at pH 7.4 and 37 °C in 6h. a) $ZnSO_4$; b) $CaSO_4$; c) $MgCl_2$.

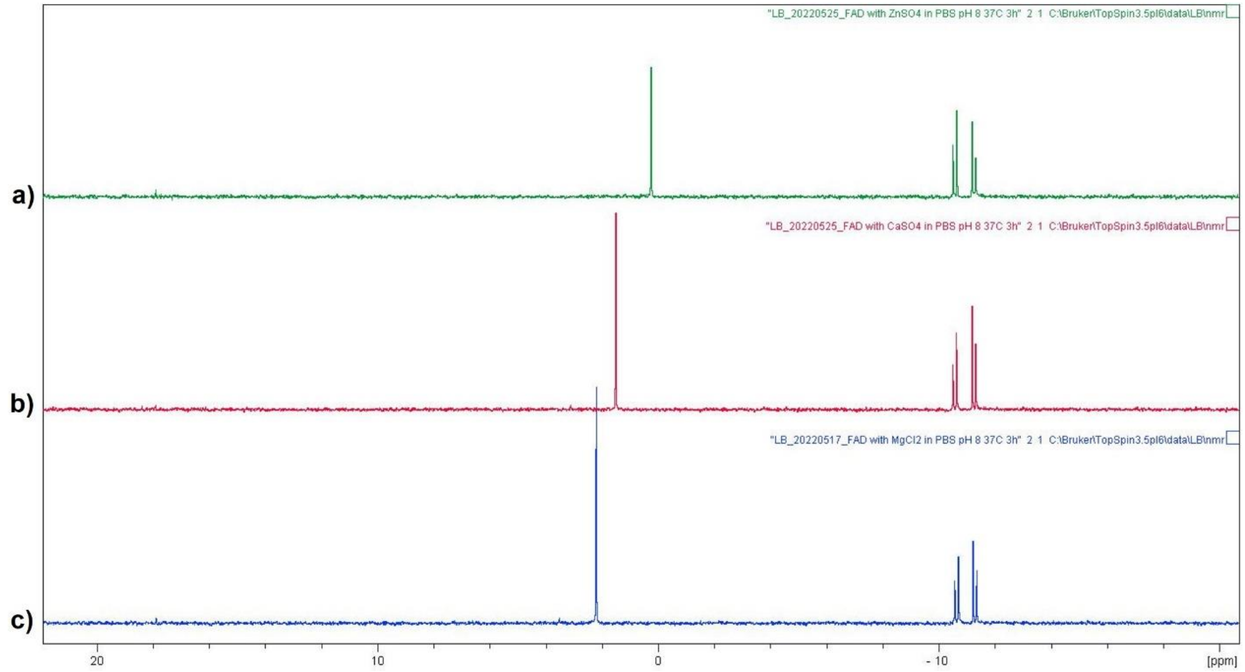

Figure S15. ^{31}P NMR (10% D_2O in H_2O, 161.1 MHz, T=25°C) of the reactions of FAD (10 mM) to form cFMN in PBS (50 mM) as a function of divalent cations (10 mM) at pH 8 and 37 °C in 3h. a) $ZnSO_4$; b) $CaSO_4$; c) $MgCl_2$.

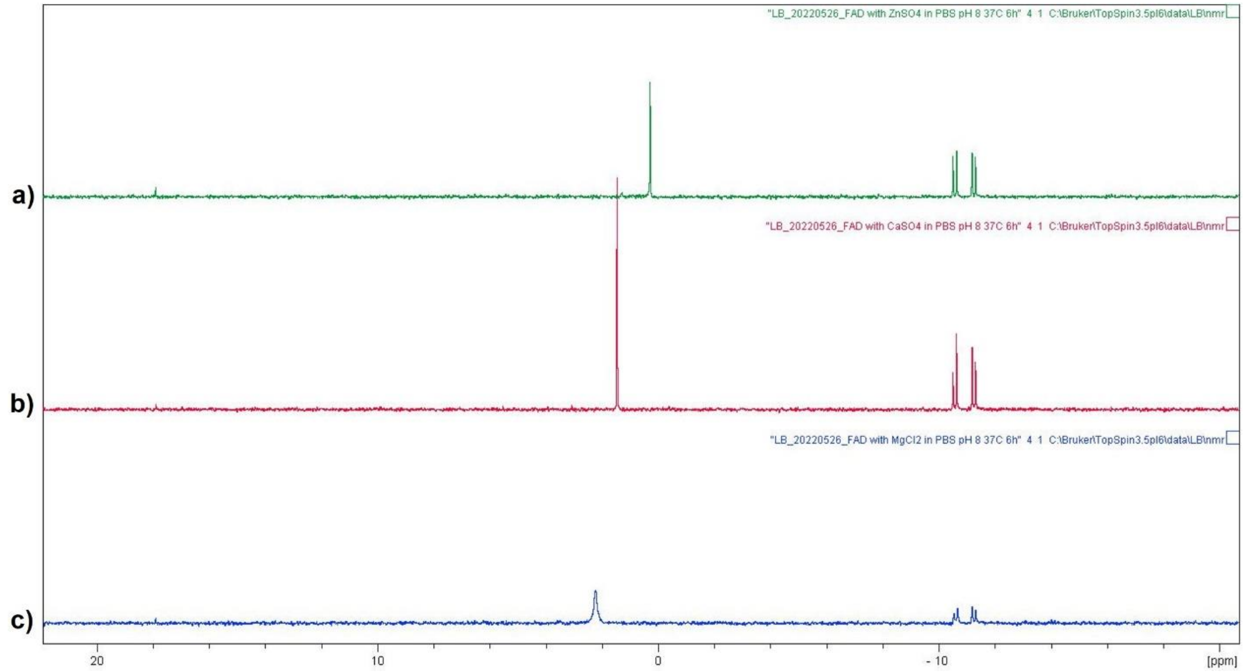

Figure S16. ^{31}P NMR (10% D_2O in H_2O, 161.1 MHz, T=25°C) of the reactions of FAD (10 mM) to

form cFMN in PBS (50 mM) as a function of divalent cations (10 mM) at pH 8 and 37 °C in 6h. a) $ZnSO_4$; b) $CaSO_4$; c) $MgCl_2$.

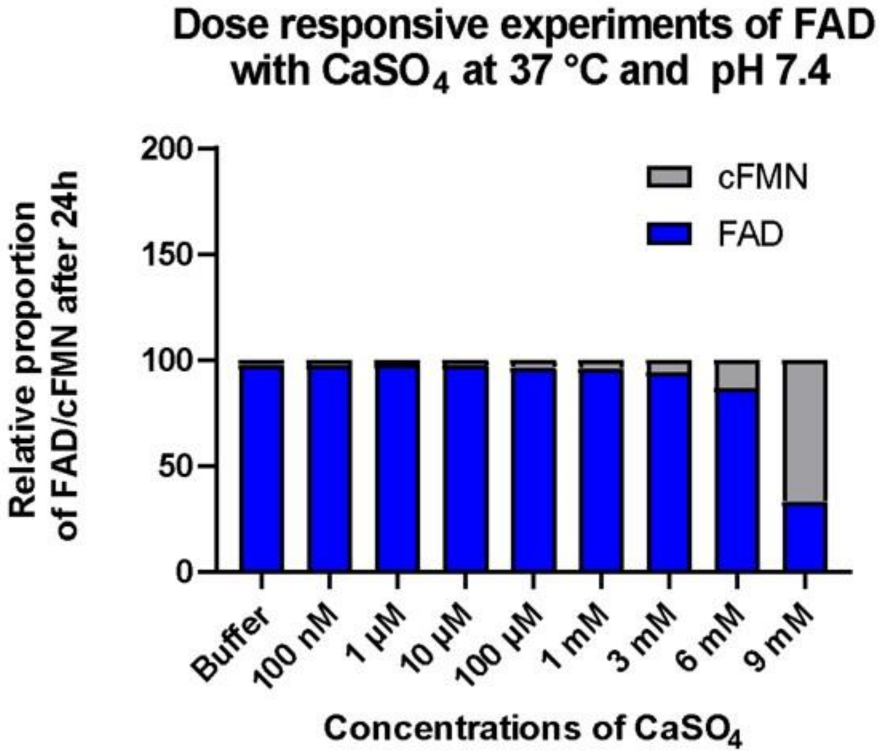

Figure S17. Reactions of FAD (10 mM) in Tris-HCl (50 mM, pH 7.4, 37°C) to form cFMN in the presence of different concentrations of $CaSO_4$ in 24h.

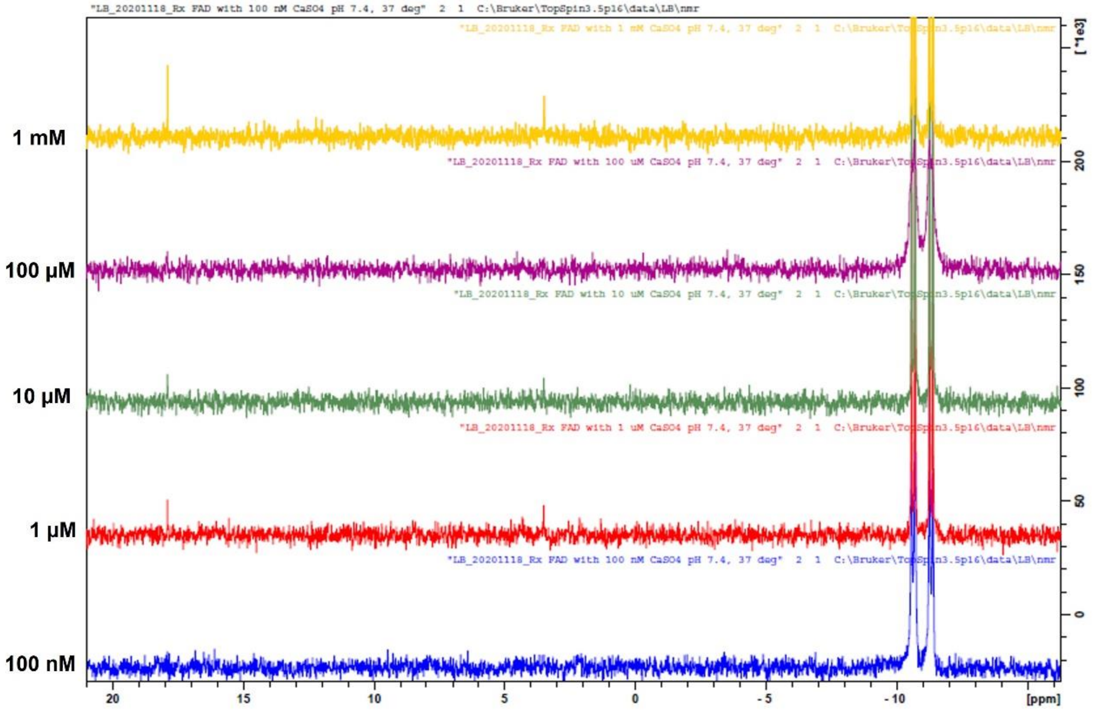

Figure S18. ^{31}P NMR (10% D_2O in H_2O, 161.1 MHz, T=25°C) of the reactions of FAD (10 mM) in Tris-HCl (50 mM, pH 7.4, 37°C) to form cFMN in the presence of different concentrations of $CaSO_4$ in 24h.

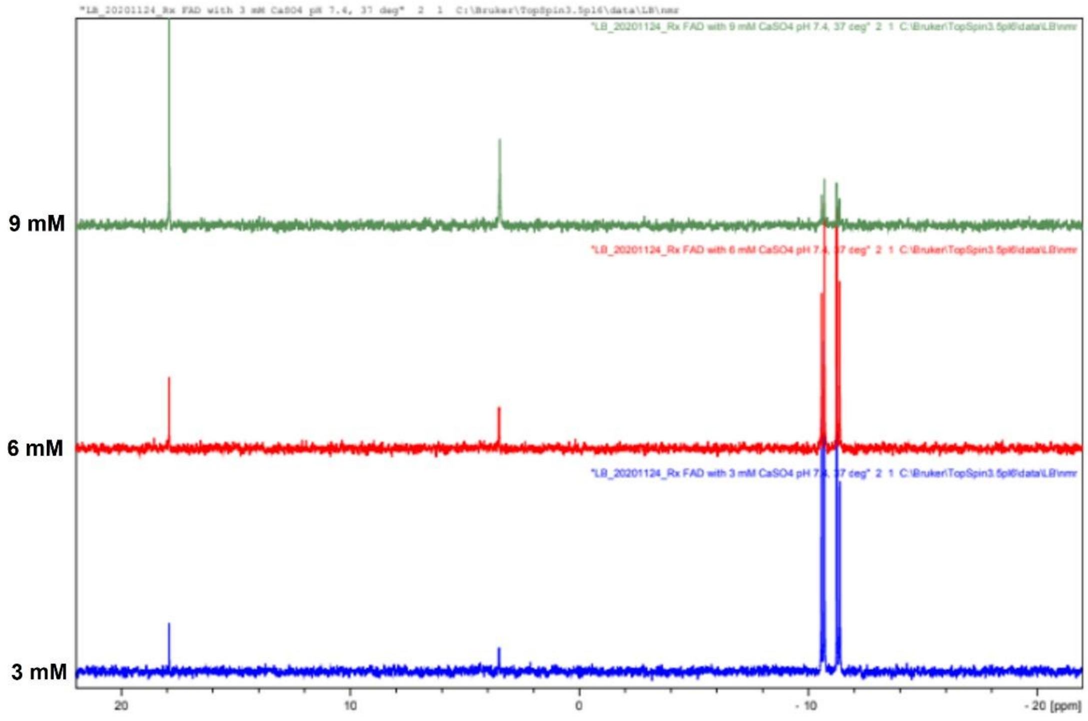

Figure S19. ^{31}P NMR (10% D_2O in H_2O, 161.1 MHz, T=25°C) of the reactions of FAD (10 mM) in Tris-HCl (50 mM, pH 7.4, 37°C) to form cFMN in the presence of different concentrations of $CaSO_4$ in 24h.

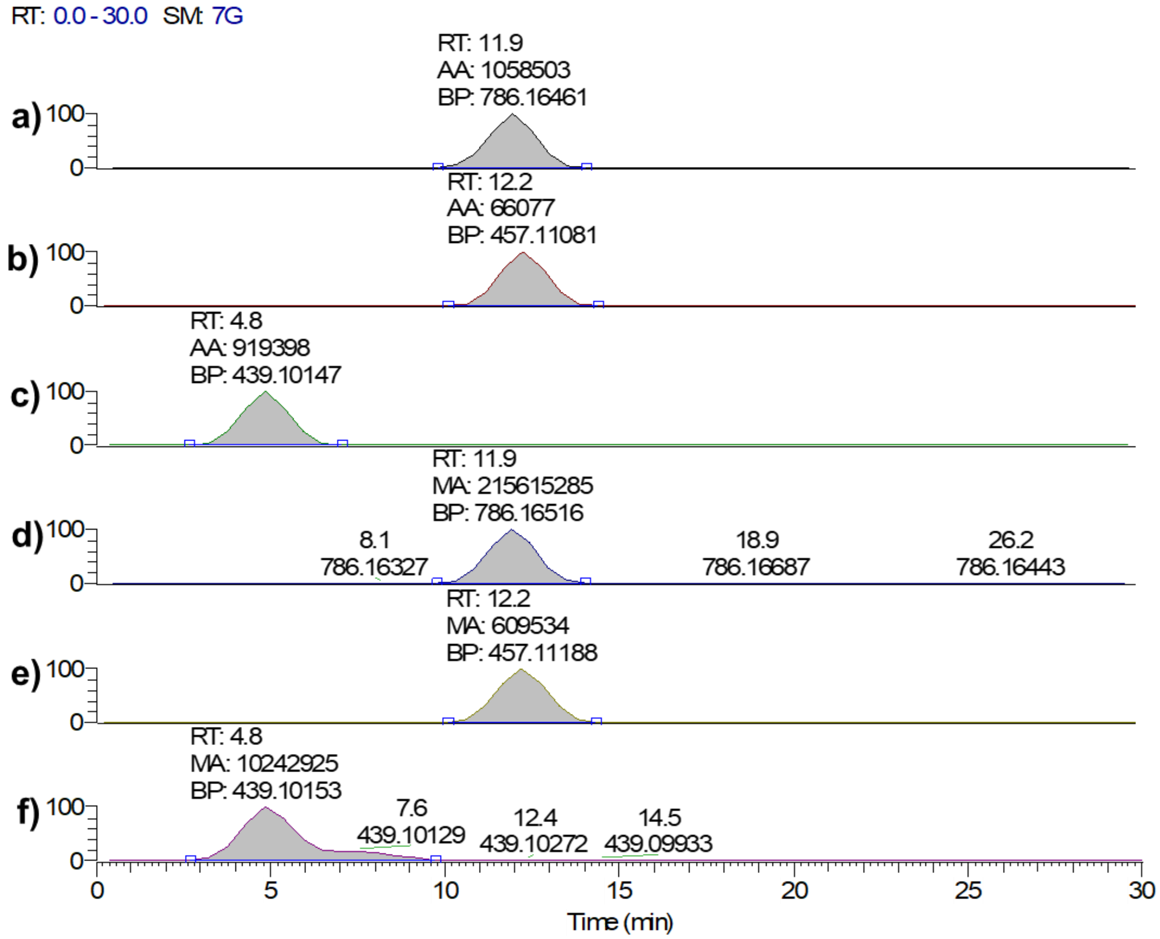

Figure S20. HILIC LC-MS chromatograms of the measurements (AUC values) of: a) FAD abundance in blood sample; b) FMN abundance in blood sample; c) cFMN abundance in blood sample d) 200 fold increase in FAD abundance in FAD-spiked blood sample; e) 9 fold increase in FMN abundance detected in the FAD-spiked blood sample; f) 11 fold increase in cFMN abundance detected in the FAD-spiked blood sample. The blood (200 µL) was spiked with 5 nmol of FAD before extraction.

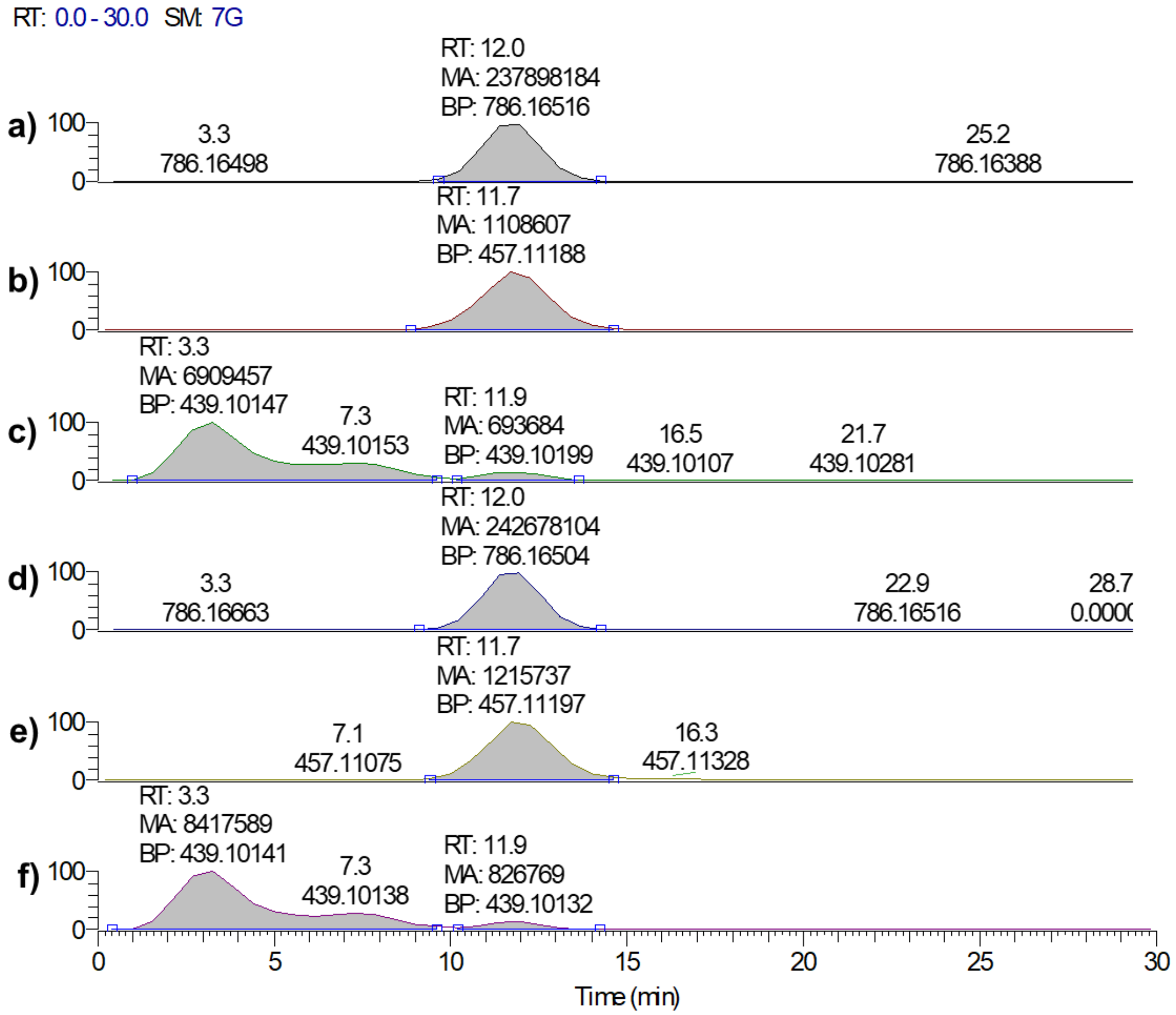

Figure S21. HILIC LC-MS chromatograms of the measurements (AUC values) of: a) Abundance of FAD standard sample following extraction; b) FMN generated from FAD standard sample following extraction or from in source fragmentation; c) cFMN generated from FAD standard sample following extraction d) FAD abundance measured in a non-extracted FAD sample; e) FMN measured in a non-extracted FAD standard sample; f) cFMN measured in a non-extracted FAD sample. 5 nmol of FAD had been added to 200 μL H_2O and extracted.

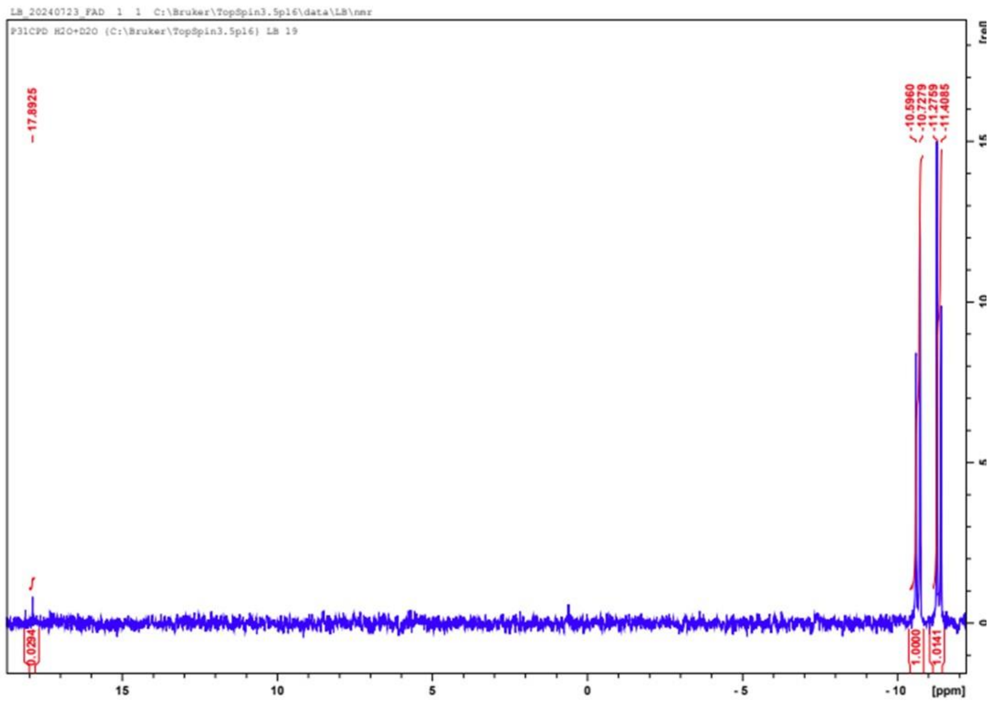

Figure S22. 31P NMR (10% D2O in H2O, 161.1 MHz, T=25 °C) of FAD showing cFMN contamination at 17.9 ppm.

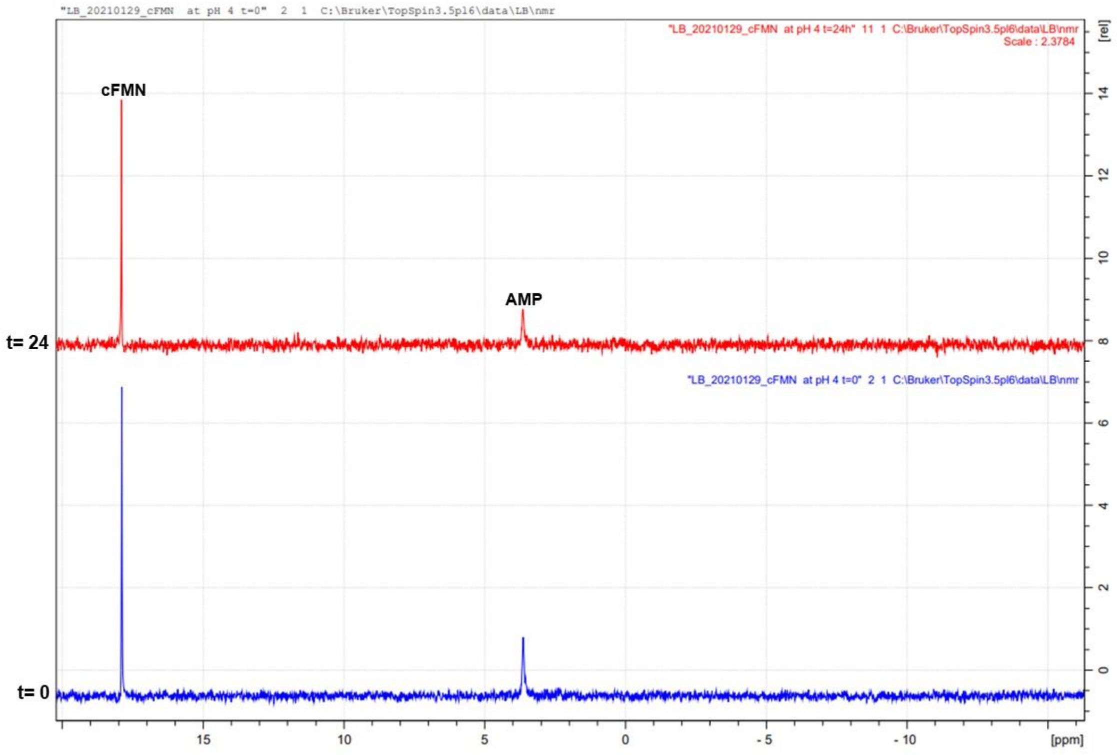

Figure S23. ^{31}P NMR (10% D_2O in H_2O, 161.1 MHz, T=25 °C) of cFMN and AMP incubated in water at pH 4 and 37 °C.

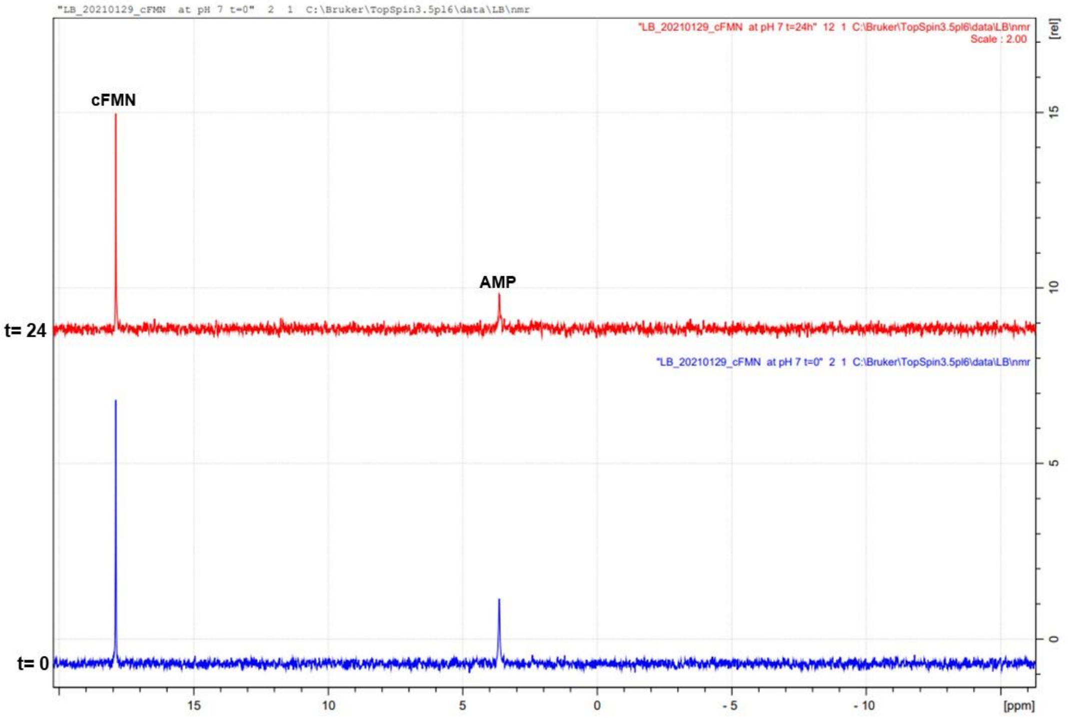

Figure S24. ^{31}P NMR (10% D_2O in H_2O, 161.1 MHz, T=25 °C) of cFMN and AMP incubated in water at pH 7 and 37 °C.

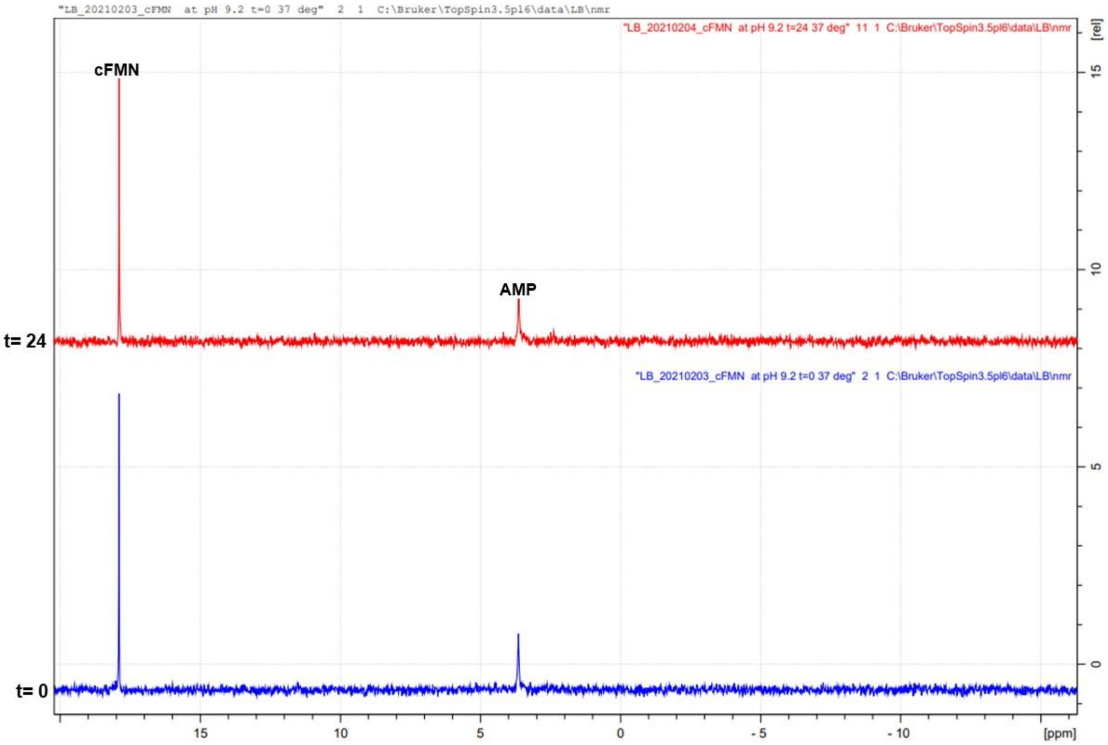

Figure S25. ^{31}P NMR (10% D_2O in H_2O, 161.1 MHz, T=25 °C) of cFMN and AMP incubated in water at pH 9.2 and 37 °C.

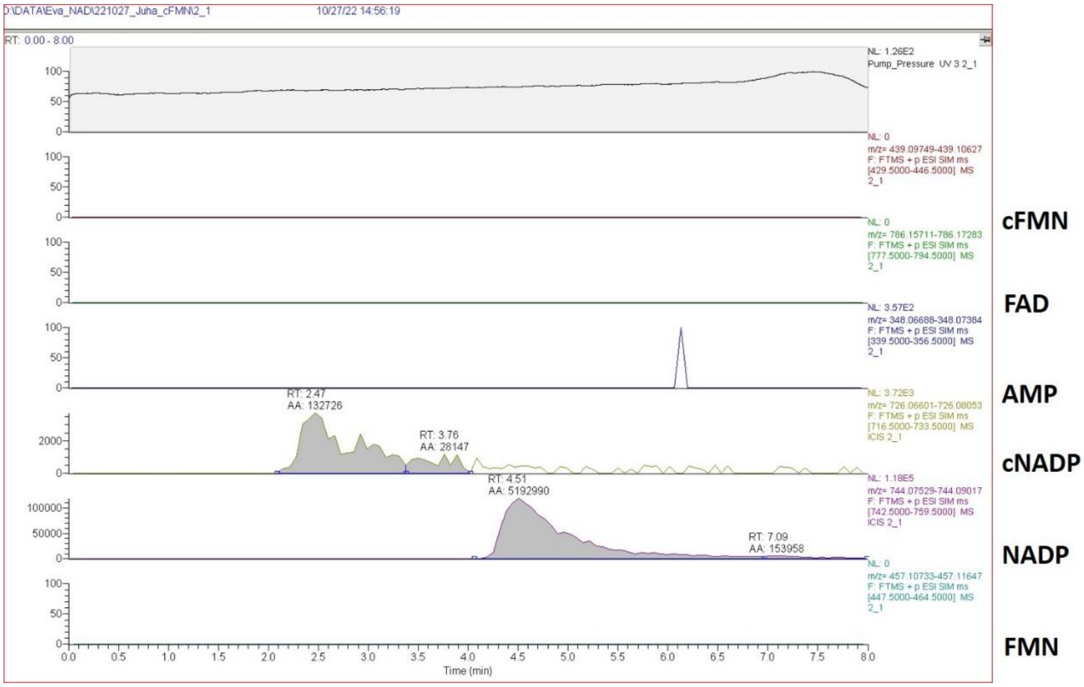

Figure S26. Mass Spectrometry chromatograms of the reaction of $cNADP^+$ by CNPase to form $NADP^+$.

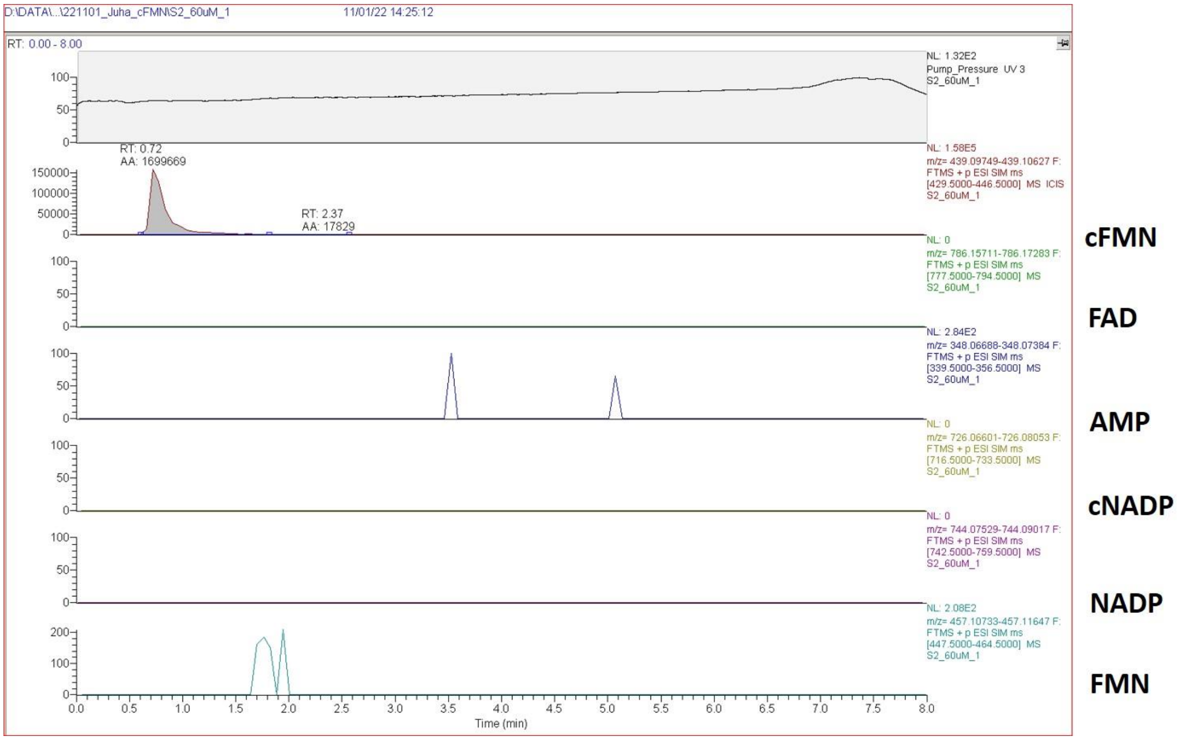

Figure S27. Mass Spectrometry chromatograms of the reaction of cFMN by CNPase to form FMN.

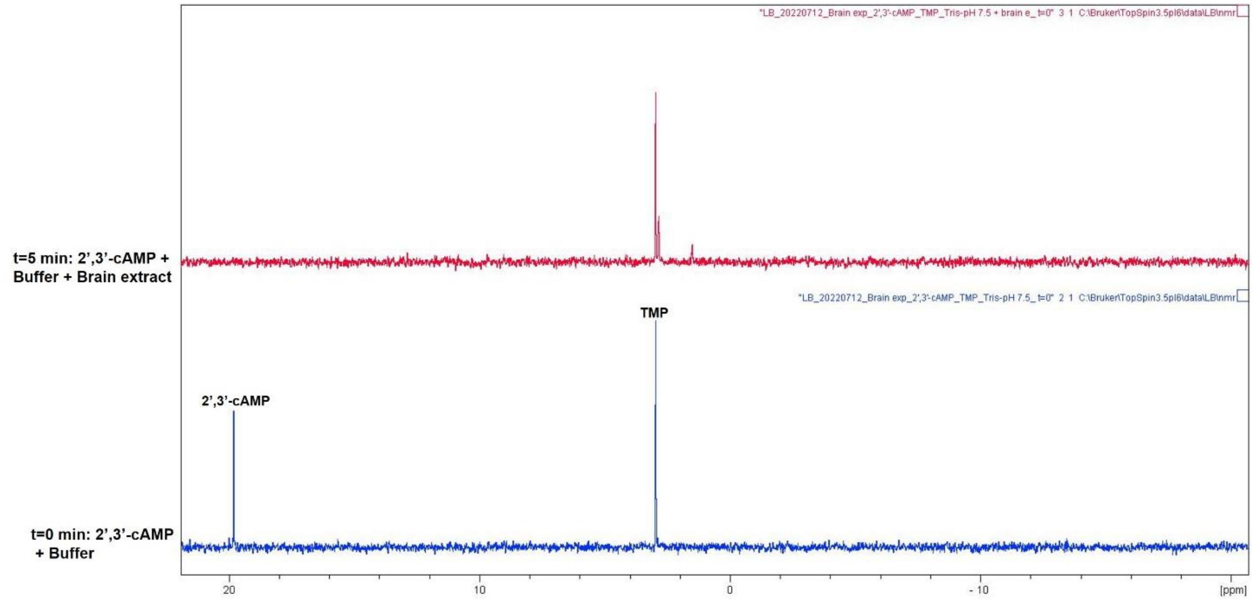

Figure S28. Brain-rich protein extracts (2.5 mg/mL) of mice tissue that hydrolyze 2′,3′-cAMP (2

mM). The reactions were monitored by ^{31}P NMR (ns =40). Trimethyl phosphate (TMP; 2 mM) has used as internal standard.

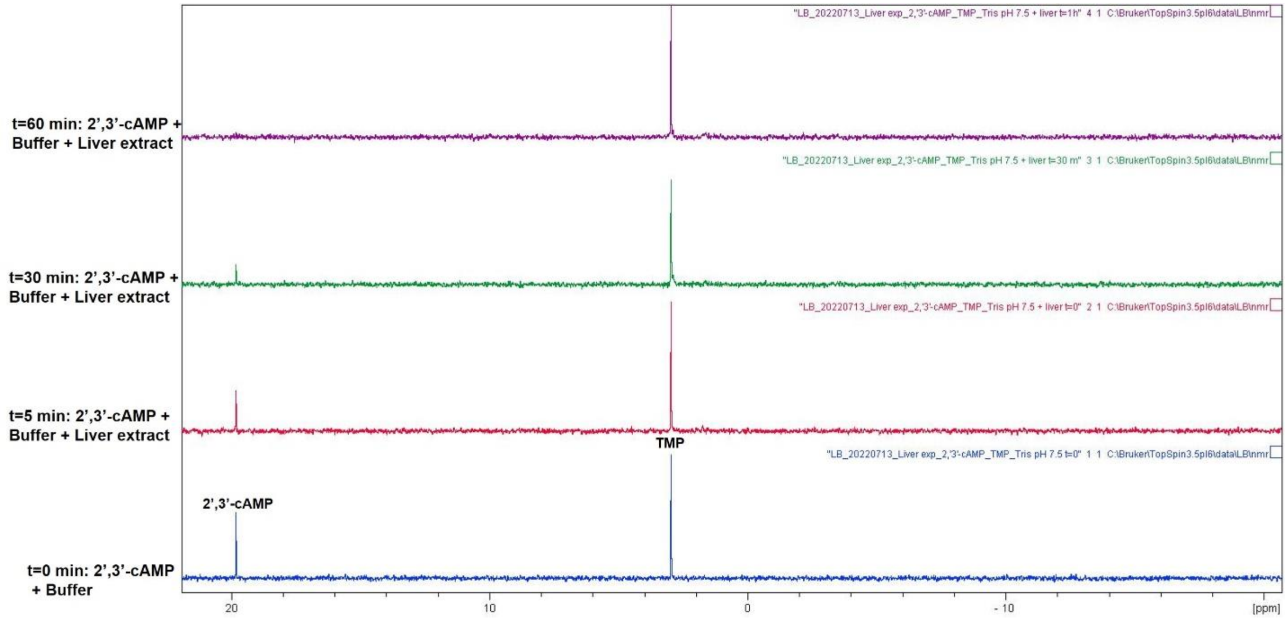

Figure S29. Liver-rich protein extracts (2.5 mg/mL) of mice tissue that hydrolyze 2′,3′-cAMP (2 mM). The reactions were monitored by ^{31}P NMR (ns =40). Trimethyl phosphate (TMP; 2 mM) has used as internal standard.

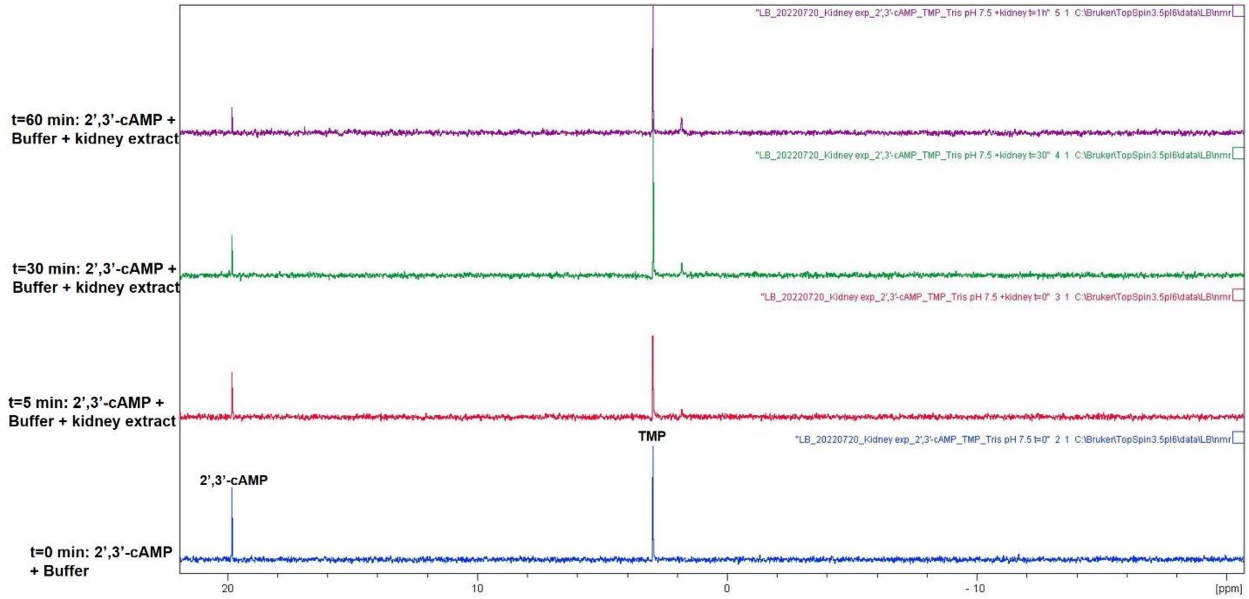

Figure S30. Kidney-rich protein extracts (2.5 mg/mL) of mice tissue that hydrolyze 2′,3′-cAMP (2 mM). The reactions were monitored by ^{31}P NMR (ns =40). Trimethyl phosphate (TMP; 2 mM) has used as internal standard.

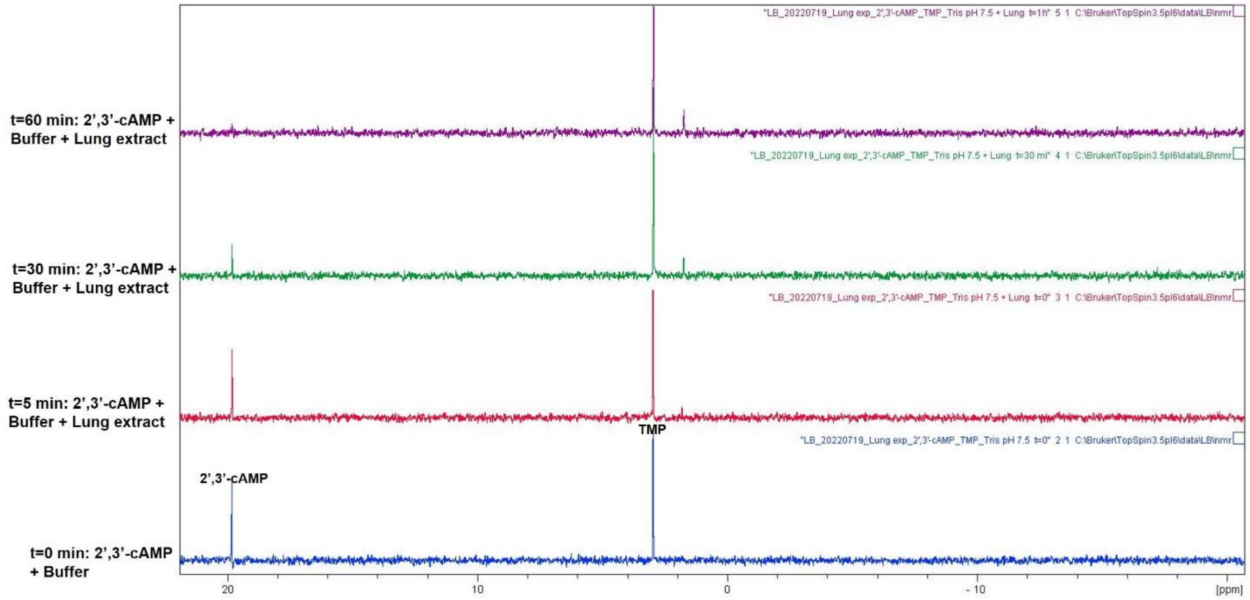

Figure S31. Lung-rich protein extracts (2.5 mg/mL) of mice tissue that hydrolyze 2′,3′-cAMP (2 mM). The reactions were monitored by ^{31}P NMR (ns =40). Trimethyl phosphate (TMP; 2 mM) has used as internal standard.

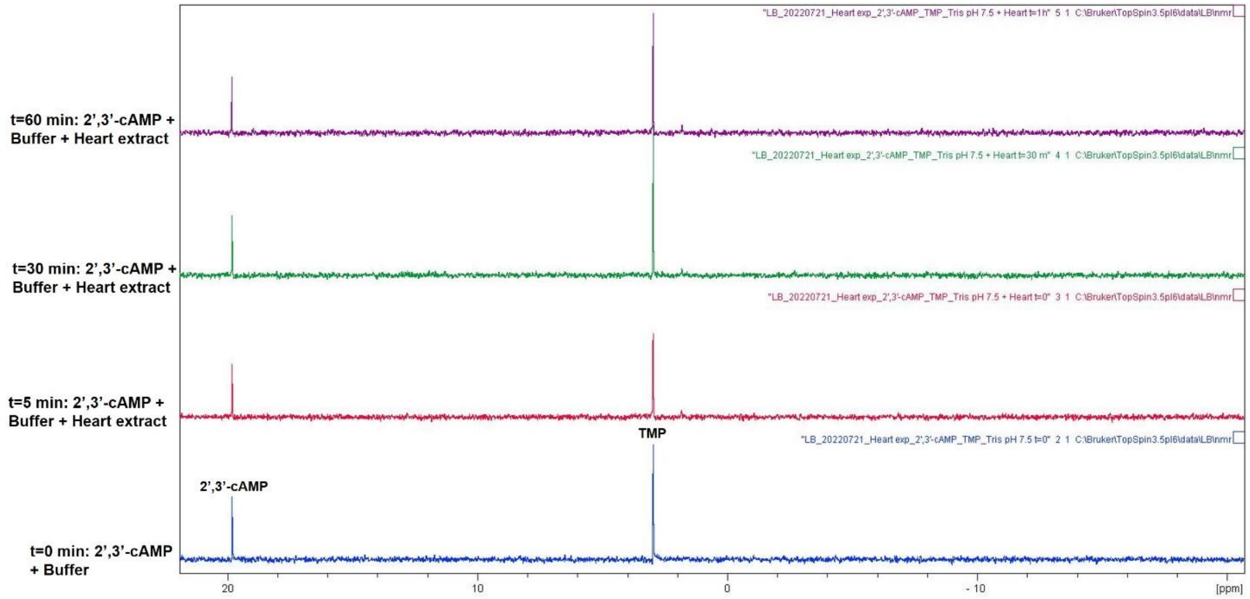

Figure S32. Heart-rich protein extracts (2.5 mg/mL) of mice tissue that hydrolyze 2′,3′-cAMP (2 mM). The reactions were monitored by ^{31}P NMR (ns =40). Trimethyl phosphate (TMP; 2 mM) has used as internal standard.

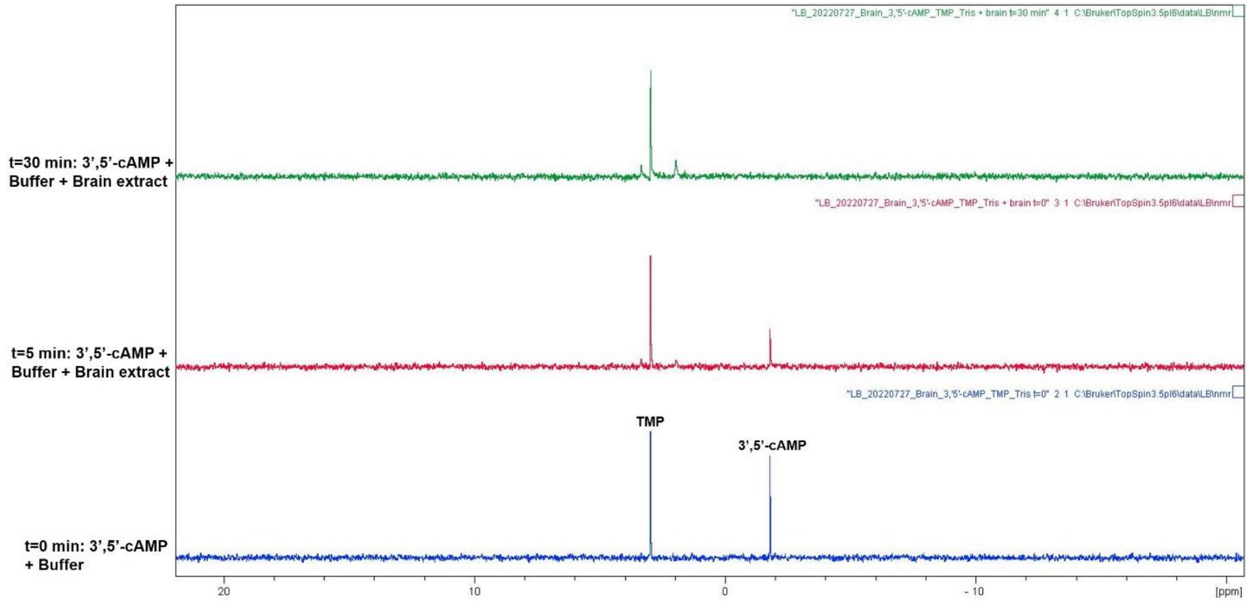

Figure S33. Brain-rich protein extracts (2.5 mg/mL) of mice tissue that hydrolyze 3′,5′-cAMP (2 mM). The reactions were monitored by ^{31}P NMR (ns =40). Trimethyl phosphate (TMP; 2 mM) has used as internal standard.

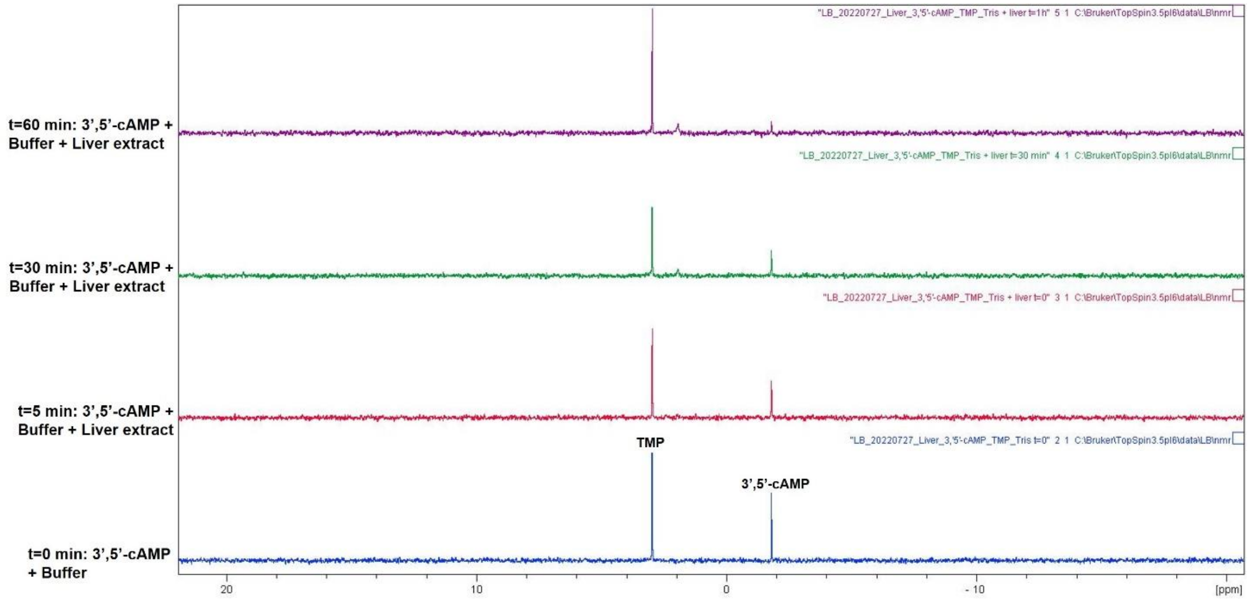

Figure S34. Liver-rich protein extracts (2.5 mg/mL) of mice tissue that hydrolyze 3′,5′-cAMP (2 mM). The reactions were monitored by ^{31}P NMR (ns =40). Trimethyl phosphate (TMP; 2 mM) has used as internal standard.

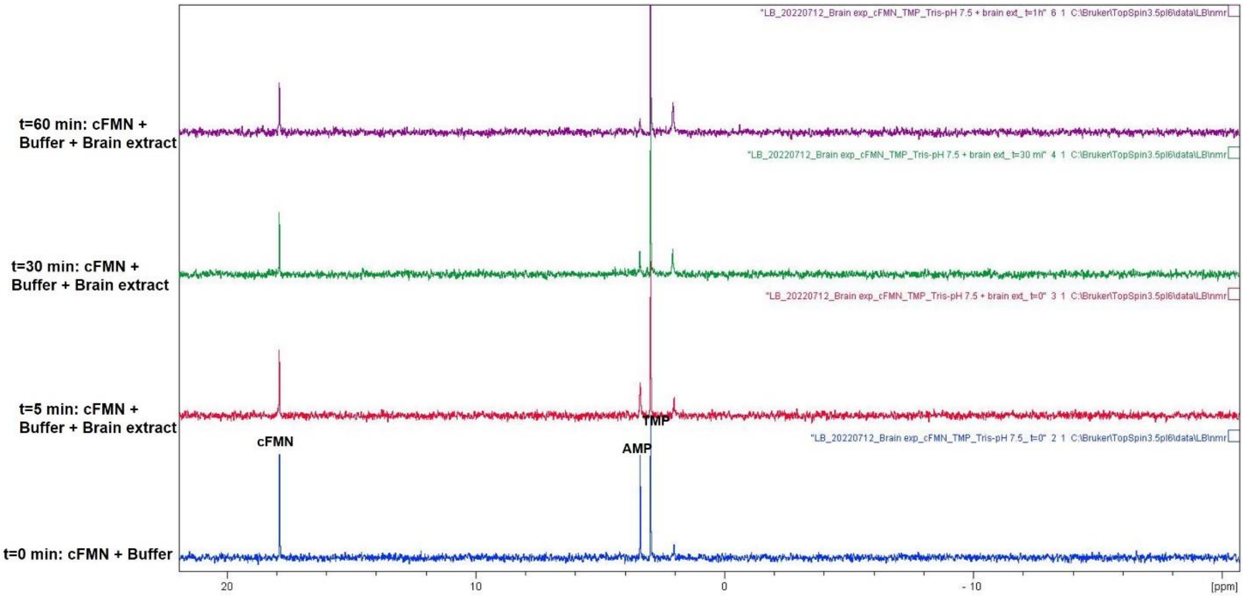

Figure S35. Brain-rich protein extracts (2.5 mg/mL) of mice tissue that hydrolyze cFMN (2 mM). The reactions were monitored by ^{31}P NMR (ns =40). Trimethyl phosphate (TMP; 2 mM) has used as internal standard.

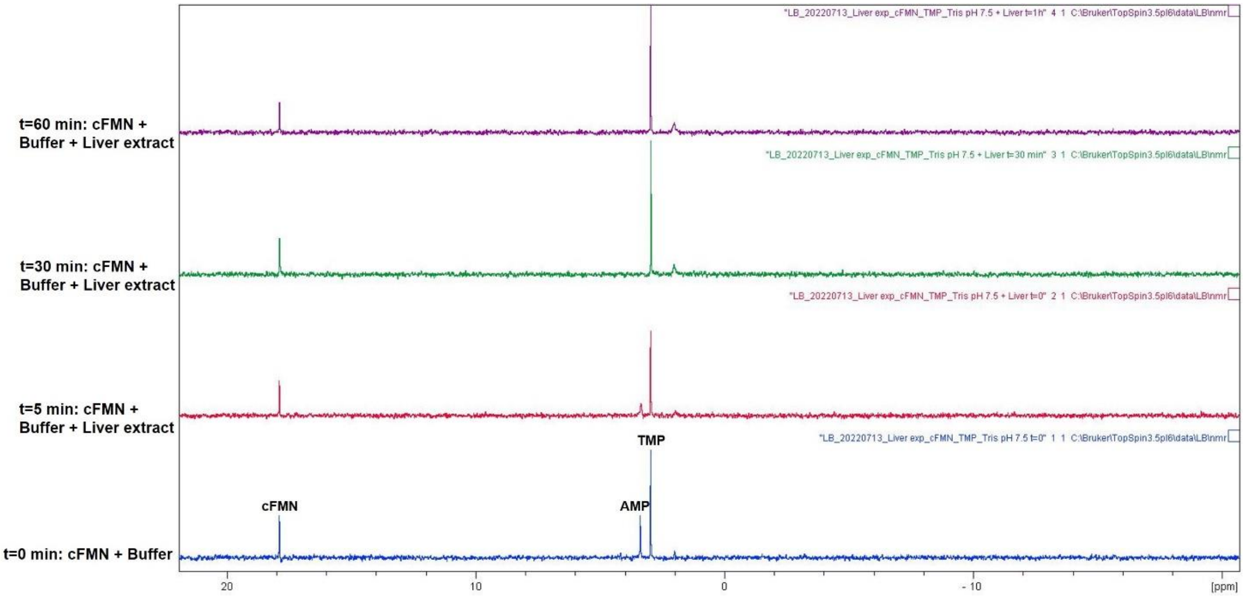

Figure S36. Liver-rich protein extracts (2.5 mg/mL) of mice tissue that hydrolyze cFMN (2 mM). The reactions were monitored by ^{31}P NMR (ns =40). Trimethyl phosphate (TMP; 2 mM) has used as internal standard.

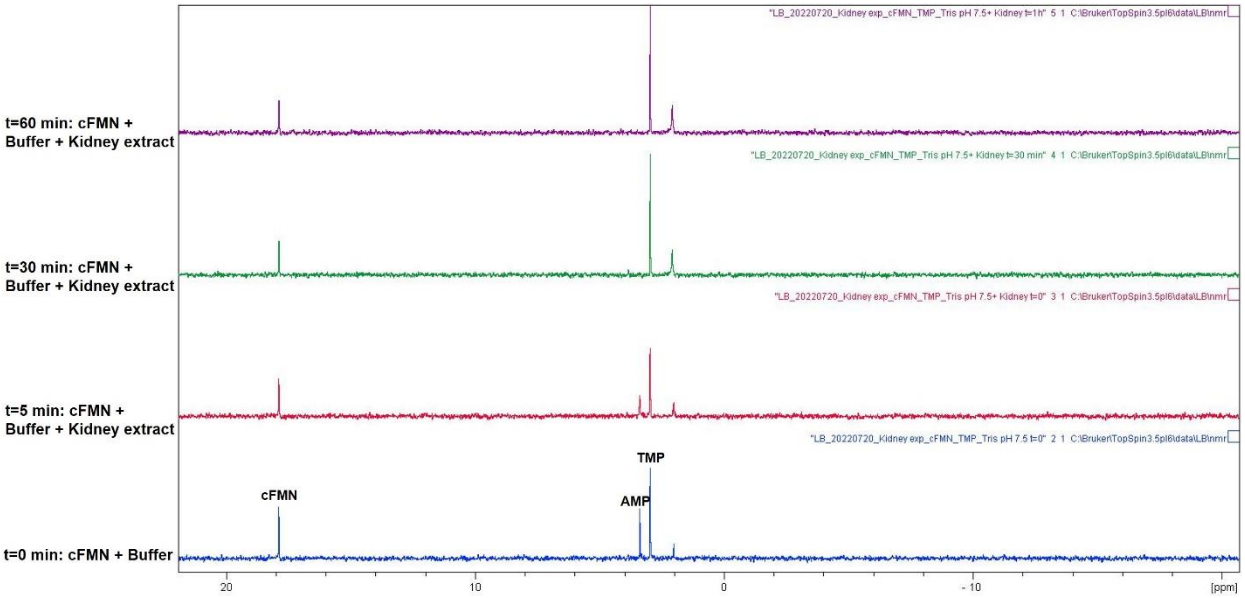

Figure S37. Kidney-rich protein extracts (2.5 mg/mL) of mice tissue that hydrolyze cFMN (2 mM). The reactions were monitored by ^{31}P NMR (ns =40). Trimethyl phosphate (TMP; 2 mM) has used as internal standard.

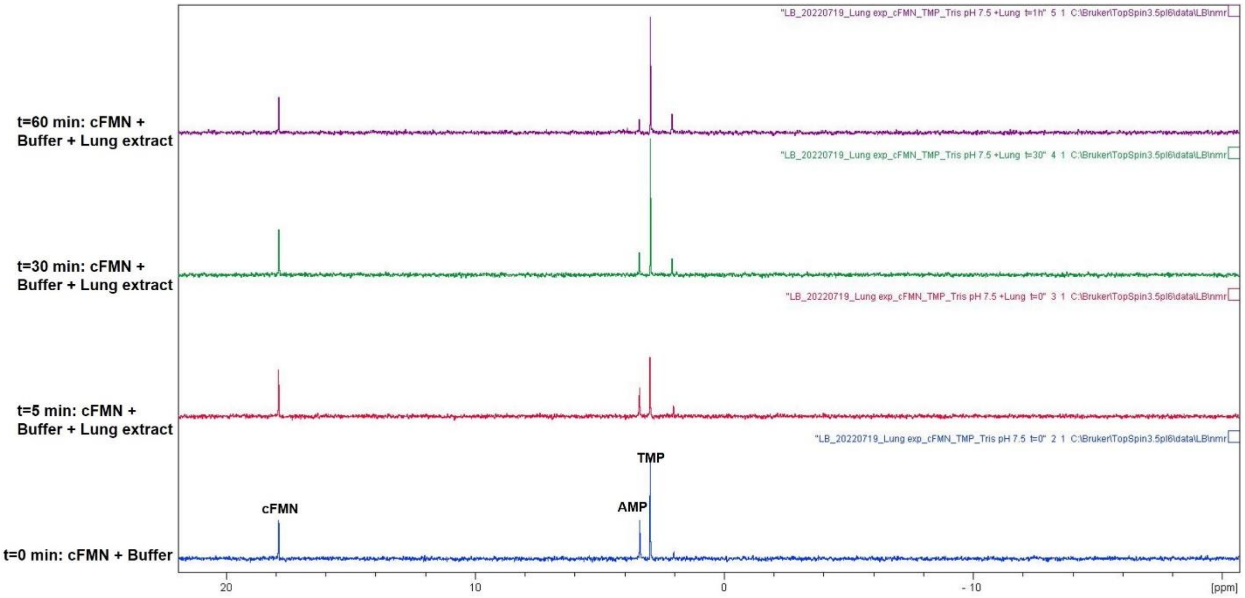

Figure S38. Lung-rich protein extracts (2.5 mg/mL) of mice tissue that hydrolyze cFMN (2 mM). The reactions were monitored by ^{31}P NMR (ns =40). Trimethyl phosphate (TMP; 2 mM) has used as internal standard.

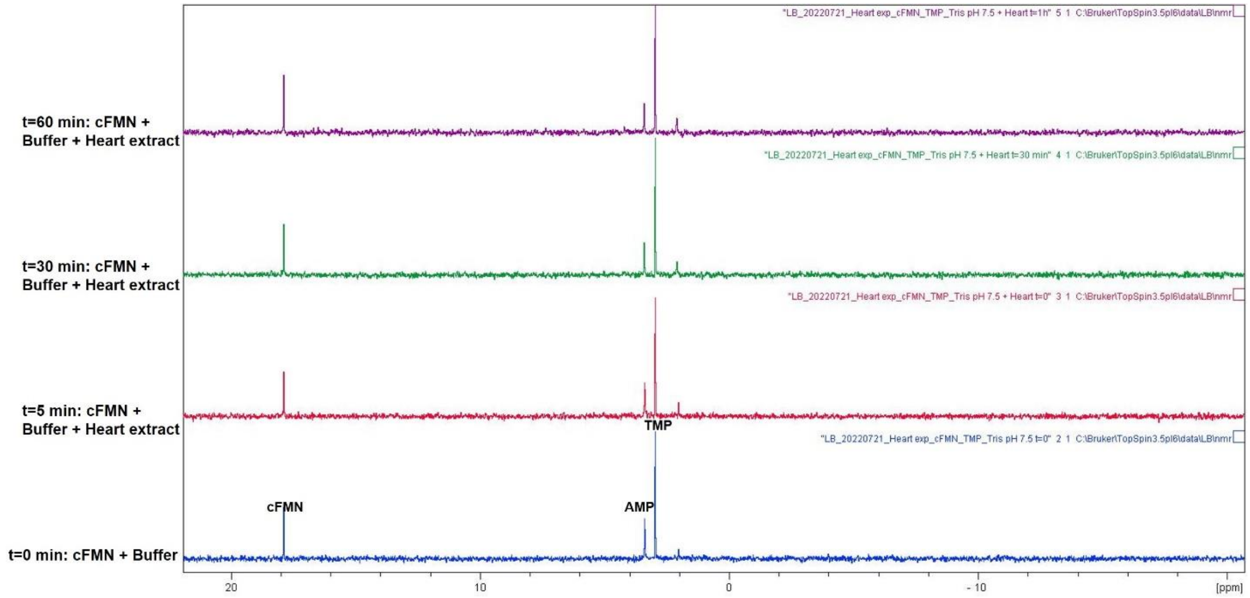

Figure S39. Heart-rich protein extracts (2.5 mg/mL) of mice tissue that hydrolyze cFMN (2 mM). The reactions were monitored by ^{31}P NMR (ns =40). Trimethyl phosphate (TMP; 2 mM) has used as internal standard.
